# mTORC2-AKT signaling to ATP-citrate lyase drives brown adipogenesis and de novo lipogenesis

**DOI:** 10.1038/s41467-020-14430-w

**Published:** 2020-01-29

**Authors:** C. Martinez Calejman, S. Trefely, S. W. Entwisle, A. Luciano, S. M. Jung, W. Hsiao, A. Torres, C. M. Hung, H. Li, N. W. Snyder, J. Villén, K. E. Wellen, D. A. Guertin

**Affiliations:** 1 Program in Molecular Medicine, University of Massachusetts Medical School, Worcester, MA 01605 USA; 2 Department of Cancer Biology, University of Pennsylvania, Philadelphia, PA 19104 USA; 3 Abramson Family Cancer Research Institute, University of Pennsylvania, Philadelphia, PA 19104 USA; 4 AJ Drexel Autism Institute, Drexel University, Philadelphia, PA 19104 USA; 5 Department of Genome Sciences, University of Washington, Seattle, WA 98195 USA; 6 Program in Molecular and Cellular Biology, University of Washington, Seattle, WA 98195 USA; 7 Department of Molecular, Cell and Cancer Biology, University of Massachusetts Medical School, Worcester, MA 01605 USA

**Keywords:** Kinases, Proteomics

## Abstract

mTORC2 phosphorylates AKT in a hydrophobic motif site that is a biomarker of insulin sensitivity. In brown adipocytes, mTORC2 regulates glucose and lipid metabolism, however the mechanism has been unclear because downstream AKT signaling appears unaffected by mTORC2 loss. Here, by applying immunoblotting, targeted phosphoproteomics and metabolite profiling, we identify ATP-citrate lyase (ACLY) as a distinctly mTORC2-sensitive AKT substrate in brown preadipocytes. mTORC2 appears dispensable for most other AKT actions examined, indicating a previously unappreciated selectivity in mTORC2-AKT signaling. Rescue experiments suggest brown preadipocytes require the mTORC2/AKT/ACLY pathway to induce PPAR-gamma and establish the epigenetic landscape during differentiation. Evidence in mature brown adipocytes also suggests mTORC2 acts through ACLY to increase carbohydrate response element binding protein (ChREBP) activity, histone acetylation, and gluco-lipogenic gene expression. Substrate utilization studies additionally implicate mTORC2 in promoting acetyl-CoA synthesis from acetate through acetyl-CoA synthetase 2 (ACSS2). These data suggest that a principal mTORC2 action is controlling nuclear-cytoplasmic acetyl-CoA synthesis.

## Abstract (web summary)

mTORC2 activates Akt, a regulator of cell growth and metabolism, however, the role of mTORC2 in adipocytes is incompletely understood. Here the authors report that a mTORC2-Akt axis specifically activates ACLY to promote lipid synthesis and histone acetylation during brown adipocyte differentiation.

## Introduction

A cell’s capacity to sense and respond to nutrients is essential for metabolic homeostasis and losing this flexibility is a hallmark of diabetes and cancer. The mammalian target of rapamycin (mTOR) kinase is a master regulator of intracellular metabolism that responds to insulin signaling and nutrient availability. Its biochemical functions are split between two complexes called mTOR complex 1 (mTORC1), which contains the essential subunit Raptor, and mTOR complex 2 (mTORC2), which contains the essential subunits Rictor and Sin1^1,2^. Amino acids and insulin stimulate mTORC1 to promote protein, lipid, and nucleic acid synthesis through well-defined downstream signaling networks. mTORC2 promotes glycolysis and lipid synthesis; however, its regulation and downstream mechanisms are less well defined.

The best understood biochemical function of mTORC2 is to phosphorylate the hydrophobic motif (HM) site in AKT (S473 in AKT1; S474 in AKT2; S472 in AKT3)^3^. HM phosphorylation cooperates with 3-phosphoinositide-dependent kinase 1 (PDK1)-dependent phosphorylation in the T-loop (T308 in AKT1; T309 in AKT2; T307 in AKT3) to maximally stimulate AKT catalytic activity^4,5^. Growth factors facilitate AKT phosphorylation by stimulating AKT and PDK1 recruitment to the plasma membrane through pleckstrin homology (PH) domains, which bind phosphatidylinositol 3,4,5-triphosphate (PIP3). mTORC2 and PDK1 additionally co-regulate the related AGC family kinases SGK and PKCα^6–9^. These substrates lack PH domains, thus their interaction with mTORC2 and PDK1 is likely facilitated by a different mechanism^4^. However, the temporal and spatial regulation of mTORC2 signaling remains poorly understood.

Many models show mTORC2 as an obligatory AKT activator^1,5^; however, its exact function in downstream AKT signaling in vivo remains elusive because, in many tissue-specific conditional *Rictor* knockout (KO) models, mTORC2 loss has seemingly minimal-to-no effect on the phosphorylation of several AKT substrates^2,10^. For example, conditionally deleting *Rictor* in brown adipose tissue (BAT) (e.g., with *ucp1-cre* or *myf5-cre*) or in white adipose tissue (WAT) (with *adiponectin-cre*) ablates AKT HM phosphorylation without obviously impairing AKT activity toward classic substrates, including PRAS40, TSC2, GSK3β, AS160, and FoxO1^10–14^. Yet, despite seemingly normal AKT signaling, *Rictor* loss profoundly downregulates transcription of ATP citrate lyase (ACLY), acetyl-CoA carboxylase (ACC), and fatty acid synthase (FASN), which catalyze de novo lipogenesis (DNL)^11,12^. *Rictor* loss also reduces glucose uptake and inhibits the expression of ChREBPβ^12,13^, which is a constitutively active isoform of the carbohydrate response element binding protein (ChREBP) transcription factor that stimulates carbohydrate and lipid metabolic gene expression^15^. Similarly, inducible deletion of *Rictor* in brown preadipocytes has seemingly no effect on downstream AKT signaling, yet renders these cells incapable of differentiating in vitro^11^. Nevertheless, expressing recombinant AKT1 containing a phospho-mimetic S473 residue in *Rictor*-deficient preadipocytes rescues differentiation. The latter observation suggests that mTORC2 may be more essential for some AKT actions that are required for brown adipocyte differentiation and *de novo* lipogenesis, but dispensable for many others.

ACLY cleaves extra-mitochondrial citrate to generate acetyl-CoA, which is the precursor for glucose-dependent de novo lipid and cholesterol biosynthesis. Acetyl-CoA is also used to acetylate lysine residues on histones and metabolic proteins to regulate gene expression and enzyme activity, respectively. ACLY serine 455 lies within a basophilic phosphorylation motif (RxxS) that is similar to the AKT consensus motif (RxRxxS/T). Phosphorylation of this site stimulates ACLY activity; however, the serine 455 kinase remains controversial as AKT^16^, PKA^17,18^, mTOR^19^, or the branched chain ketoacid dehydrogenase kinase^20^ can reportedly phosphorylate this site. Nevertheless, because ACLY functions at the interface of glucose-dependent DNL and epigenetic control of gene expression, it is poised to be a key link between hormonal signaling and carbohydrate and lipid metabolism in adipocytes.

Here we test the hypothesis that mTORC2 promotes brown adipocyte differentiation and glucose-driven DNL (gluco-lipogenesis) through a distinctly mTORC2-dependent AKT pathway. To do this, we analyzed mTORC2-regulated AKT phosphosites and metabolites by mass spectrometry. This led us to identify a subset of AKT substrates and metabolites that are regulated by mTORC2, including the enzyme ACLY and its product acetyl-CoA. We provide evidence that ACLY functions downstream of a uniquely mTORC2-dependent AKT pathway required for DNL, differentiation, histone acetylation, and ChREBPβ and gluco-lipogenic gene expression. Substrate utilization studies suggest an additional role for mTORC2 in promoting acetyl-CoA synthesis from acetate through acyl-CoA synthetase short chain family member 2 (ACSS2). These data uncover a previously unappreciated selectivity in mTORC2-dependent AKT signaling in *Rictor*-deleted brown adipocytes and reveal mTORC2’s central role in controlling nuclear–cytoplasmic acetyl-CoA synthesis.

## Results

### Stratification of AKT substrates by mTORC2 sensitivity

To test the hypothesis that some AKT substrates are more sensitive to mTORC2 loss than for others and to identify the mechanism by which mTORC2 drives brown preadipocyte differentiation, we conducted a targeted phosphoproteomics assay comparing the insulin response between *Rictor-iKO* precursor brown adipocytes (*Rictor-iKO*^*PBAs*^) and isogenic control brown preadipocytes. We designed the assay to quantify phosphopeptides encompassing regulatory phosphorylation sites on AKT, previously reported AKT and SGK substrate sites, and other potential mTORC2 effectors. *Rictor-iKO*^*PBAs*^ maintain growth-factor-stimulated AKT^T308^ phosphorylation, albeit at lower levels, and seemingly normal phosphorylation of AKT substrates [Supplementary Fig. 1A], as previously observed^11^. *Rictor-iKO*^*PBAs*^ and their vehicle-treated isogenic controls were then serum deprived, or serum deprived and then stimulated with insulin for 15 min. Phosphopeptide-enriched samples were analyzed with parallel reaction monitoring targeted mass spectrometry, a highly sensitive quantitative technique. The assay quantified 31 known AKT substrates, of which 17 increase phosphorylation at least 1.5-fold in response to insulin in wild-type (WT) cells at Benjamini–Hochberg 5% false detection rate (FDR), indicating that they are likely targets of insulin-stimulated AKT action. Of the 17 insulin-upregulated phosphopeptides, 9 contain AKT substrate motifs for which the phosphorylated residue can be localized with high confidence. The other eight contain phosphorylated residues with ambiguous localization within at most three residues away from the predicted AKT substrate site.

**Fig. 1:**
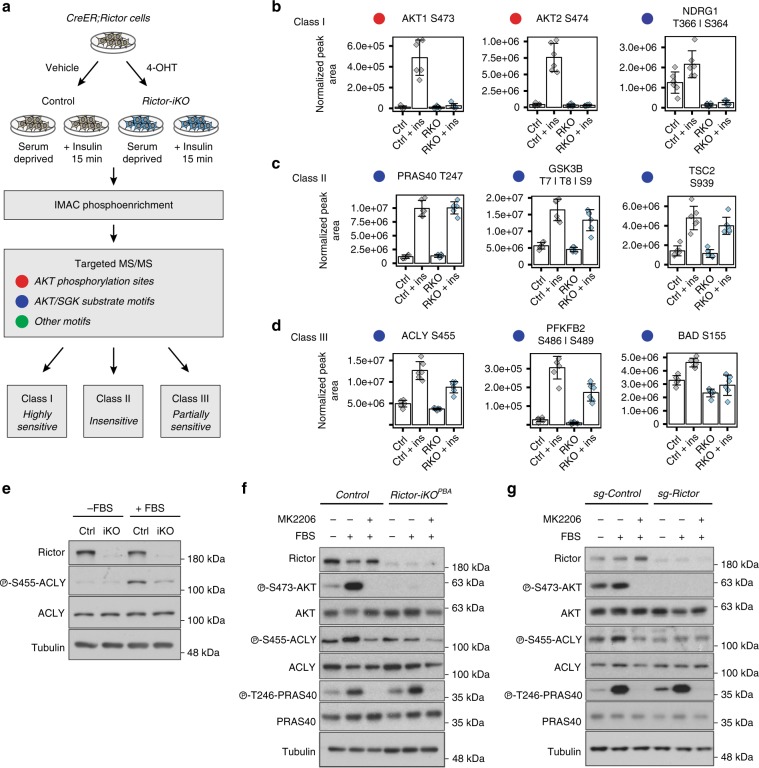
Identification of mTORC2-sensitive phosphorylation sites, including S455 in ATP citrate lyase (ACLY). **a** Phosphoproteomics work flow and analysis in *Rictor-iKO*^*PBAs*^. Colored circles correspond with the type of motifs analyzed (*n* = 6 per group). **b**, **c** Motifs were stratified by their sensitivity to mTORC2 loss as being highly sensitive Class I sites (**b**), insensitive Class II sites (**c**), or partially sensitive Class III sites (**d**). **e** Western blots of protein lysates from control and *Rictor-iKO*^*PBA*^ cells using the indicated total and phospho-specific antibodies. Cells growing in DMEM were either serum deprived (−FBS) or serum deprived and then stimulated with fresh serum for 15 min (+FBS) prior to lysis. **f** Western blots of protein lysates treated as in **e** with or without the AKT inhibitor MK2206. **g** Western blots using lysates from HEK293 cells in which *Rictor* was deleted by CRISPR/Cas9 genome editing. The AKT inhibitor MK2206 (10 μM) was administered 1 h prior to lysis in both **f** and **g**.

Based on quantitative differences between conditions, we stratified phosphopeptides into three classes, all with a 5% FDR from analysis of variance (ANOVA; see “Methods”): Class I contains phosphosites that are highly dependent upon mTORC2; Class II contains phosphosites that are insensitive to mTORC2 loss; and Class III contains phosphosites that are partially sensitive to mTORC2 loss [Fig. 1a, Supplementary Data 1]. Among the Class I peptides, we identified the known direct mTORC2 target sites pAKT1^S473^, pAKT2^S474^, and pAKT3^S472^ as insulin stimulated and highly sensitive to mTORC2 loss [Fig. 1b, Supplementary Fig. 2A]. We also identified a Class I peptide from NDRG1 with phosphorylation on either S364 or T366 that was not significantly stimulated by insulin but is highly dependent of mTORC2 [Fig. 1b]. This difference is not explained by changes to NDRG1 protein levels as measured by mass spectrometry. pNDRG1^T366^ is known to be phosphorylated by SGK, a kinase from the same family of AKT that requires mTORC2-dependent HM phosphorylation for its activation^6^. These findings confirm our ability to identify phosphorylation sites that occur with or without insulin stimulation for which mTORC2 is essential.

Among the Class II substrates, for which mTORC2 is dispensable, we identified several AKT target sites, including pPRAS40^T246^ and pGSK3β^S9^ [Fig. 1c, Supplementary Fig. 2B], validating our previously observed immunoblot analyses using phospho-specific antibodies [Supplementary Fig. 1A]^11^. Also included in Class II are pTSC2^S939^ and pTSC2^S981^, two well-known AKT phosphorylation events that stimulate the mTORC1 pathway^21^ [Fig. 1c, Supplementary Fig. 2B]. Consistently, ribosomal protein S6 peptides containing phosphorylation at S235 or S236 and at S240 (often used as an mTORC1 activity reporter) are also Class II substrates [Supplementary Fig. 2B]. We additionally identified several predicted AKT substrates that fall into Class III, showing partial sensitivity to mTORC2 loss [Fig. 1d, Supplementary Fig. 2C]. One peptide is from a region in phosphofructo kinase-2 (PFK2) containing either the annotated AKT substrate site S486 or the nearby S489, and another is from a region in ACLY containing the S455 site [Fig. 1d]. We also identify mTORC2-sensitive sites on BAD (S155) and vimentin (S39) [Supplementary Fig. 2C]. These data suggest that there is selective inhibition of AKT signaling pathways following induced *Rictor* deletion in brown preadipocytes.

Immunoblotting with commercially available phosphosite-specific antibodies independently uncovered serum and insulin-stimulated pACLY^S455^ as an AKT substrate showing mTORC2 dependency [Fig. 1e, Supplementary Fig. 1B]. We were unable to detect pPFK2^S486^ using a commercially available antibody. Importantly, pACLY^S455^ and pPRAS40^T246^ (an mTORC2-insensitive AKT substrate in our assays [Fig. 1c, f, Supplementary Fig. 1B]) are both sensitive to the AKT inhibitor MK2206 [Fig. 1f, Supplementary Fig. S1B]. This argues against an AKT compensatory kinase acting in the absence mTORC2. Serum or insulin-stimulated pACLY^S455^, but not pPRAS40^T246^, is also mTORC2 dependent in HEK293E cells deleted for *Rictor* using CRISPR/Cas9, while both sites are sensitive to MK2206 [Fig. 1g, Supplementary Fig. 1C]. We also expressed recombinant Rictor in the *Rictor*-deleted HEK293E cells to confirm that this restores ACLY^S455^ phosphorylation [Supplementary Fig. 1D]. Thus selectivity may be relevant in other *Rictor*-deleted cell types.

### mTORC2 regulates acetyl-CoA availability

**Fig. 2:**
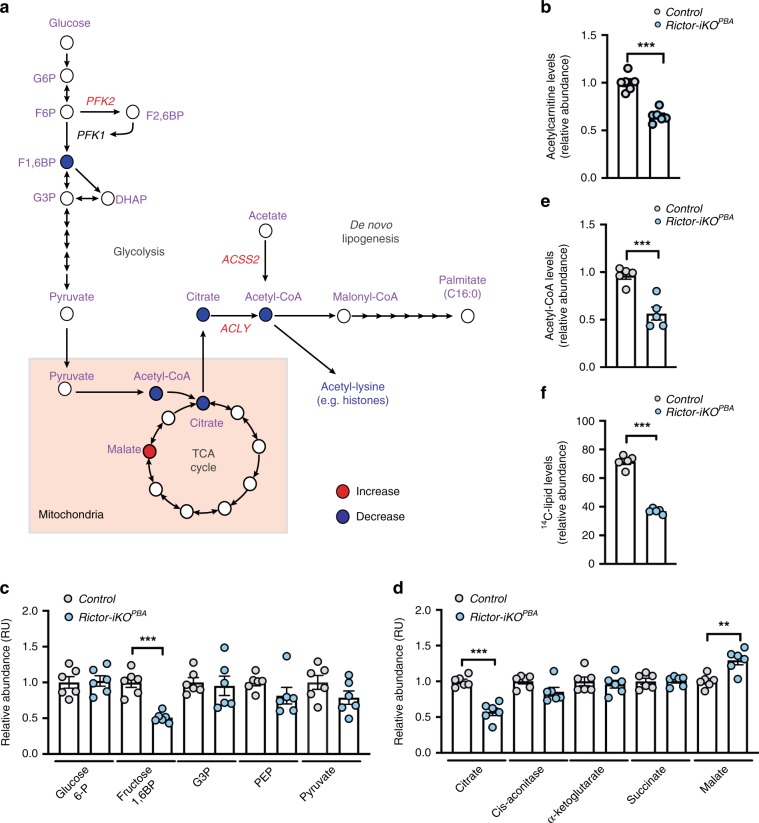
mTORC2 regulates acetyl-CoA levels and glucose-dependent fatty acid synthesis. **a** Schematic of metabolic pathways and individual metabolites regulated by mTORC2. Blue-filled circles correspond to metabolites that significantly decrease in abundance in *Rictor-iKO*^*PBA*^ cells; red-filled circles indicate a significant increase in abundance. Enzymes labeled in red are those identified in this study as being regulated by mTORC2. **b**, **c** Bar graph representations showing the average relative abundance of acetylcarnitine (**b**), glycolytic (**c**), and TCA cycle (**d**) intermediates (*n* = 6 per group). **e** Direct acetyl-CoA measurements using control and *Rictor-iKO*^*PBA*^ cells (*n* = 5 per group). **f** De novo lipogenesis assay measuring D-[U-^14^C]-glucose labeling of de novo synthesized lipids (*n* = 5 per group). Bar graphs represent mean ± SEM. **p < 0.01 and ***p < 0.001. Statistical significance was calculated by using two-tailed unpaired Student’s *t* test (**b**, **e**, **f**) or two-way ANOVA with Sidak’s test multiple comparisons (**c**, **d**).

The identification of pACLY^S455^ and pPFK2^S486/S489^ as mTORC2-sensitive AKT substrates is interesting because they function at key rate-limiting regulatory steps in the synthesis of acetyl-CoA from glucose [Fig. 2a]. ACLY cleaves mitochondria-exported citrate to generate nuclear–cytoplasmic acetyl-CoA and oxaloacetate. PFK-2 catalyzes the synthesis of fructose-2,6-bisphosphate (F2,6BP) to allosterically activate PFK-1-mediated synthesis of F1,6BP, which can also allosterically activate ACLY^17^. For both enzymes, phosphorylation at the mTORC2-sensitive site stimulates activity^16,22^, suggesting that a key mTORC2 action may be to stimulate acetyl-CoA synthesis.

We also measured the steady-state abundance of 139 additional metabolites representing intermediary metabolism, 113 of which were detected in control and *Rictor-iKO*^*PBA*s^ [Supplementary Data 2]. Consistent with an acetyl-CoA synthesis defect, acetylcarnitine (which derives from acetyl-CoA and carnitine in a fast and reversible reaction typically in equilibrium with nuclear–cytoplasmic acetyl-CoA^23^) decreases by 35.04% in *Rictor-iKO*^*BPA*s^ [Fig. 2b]. Acetylserine and pantothenic acid, both of which contribute to Coenzyme A synthesis, are also reduced by 23.6% and 12.1%, respectively [Supplementary Data 2]; the latter possibly resulting from de-repressed pantothenate kinase activity^24^. While most glycolytic intermediates are unaffected by *Rictor* loss, F1,6BP decreases by 49.1% [Fig. 2c], which could be due to the mTORC2-sensitive phosphosite in PFK-2 [Fig. 1d]. We also observe a 42.5% decrease in citrate, the tricarboxylic acid (TCA) cycle intermediate exported from the mitochondria and used by ACLY to generate acetyl-CoA [Fig. 2d]. It cannot be determined from these data as to whether this represents the intra- or extra-mitochondrial citrate fraction; nevertheless, this could reflect a metabolite-driven negative feedback effect or inherent instability. The only metabolite that significantly increases is the TCA cycle intermediate malate (by 29.6%) [Fig. 2d], possibly reflecting reduced demand for citrate to be exported. Taken together, these data support the notion that a principal mTORC2 function in intermediary metabolism is regulating acetyl-CoA levels.

To directly test whether mTORC2 regulates acetyl-CoA levels, we used stable isotope dilution liquid chromatography–mass spectrometry with ^13^C_3_^15^N_1_-labeled acyl-CoA internal standards to measure total cellular acetyl-CoA levels^25,26^. This revealed a 43.54% decrease in acetyl-CoA in *Rictor-iKO*^*BPAs*^ [Fig. 2e]. We also measured DNL from glucose via acetyl-CoA intermediates using a C^14^-glucose tracer assay. This shows a 48.1% reduction in lipid synthesis in *Rictor-iKO*^*BPA*s^ relative to controls [Fig. 2f]. Quantitative PCR and western blotting indicate that ACLY, ACC, and FASN mRNA and protein levels are similar in control and *Rictor-iKO* precursors [Supplementary Fig. 1E, F], consistent with decreased lipogenic flux. Thus integrating results from immunoblotting, phosphoproteomics, and metabolomics points to ACLY as a distinctly mTORC2-sensitive AKT substrate.

### mTORC2 drives brown adipocyte differentiation through ACLY

**Fig. 3:**
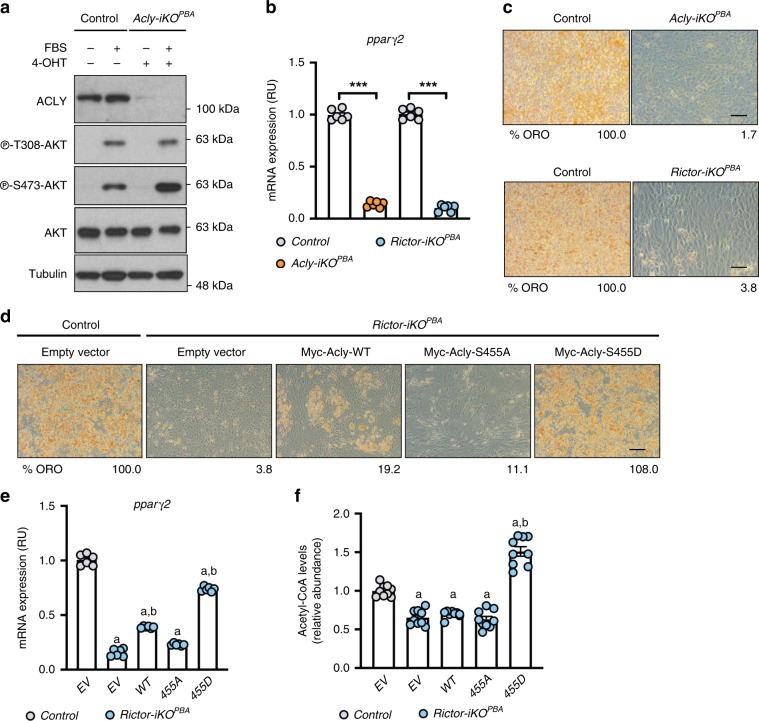
ACLY is the critical mTORC2 effector during brown adipocyte differentiation. **a** Western blot showing ablation of ACLY protein and AKT phosphorylation upon brief 4-OHT treatment of *Acly-iKO*^*PBA*^ cells. **b**, **c** *Acly-iKO*^*PBA*^ and *Rictor-iKO*^*PBA*^ cells were differentiated and *pparγ2* mRNA expression (*n* = 6 per group) (**b**) and Oil Red O (ORO) staining of lipids (**c**) were quantified at day 10 to determine differentiation efficiency. Scale bar represents 50 μm. **d**, **e** Representative differentiation rescue experiments showing ORO staining (**d**) and *Pparγ2* mRNA expression (*n* = 6 per group) (**e**) in *Rictor-iKO*^*PBA*^ cells stably expressing empty (EV), Myc-ACLY-WT, Myc-ACLY-S455A, or Myc-ACLY-455D constructs. Percentage of ORO in **d** is relative to empty vector control. Scale bar represents 50 μm. **f** Direct acetyl-CoA measurements of *Rictor-iKO*^*PBA*^ cells (*n* = 9 per group) stably expressing empty (EV), Myc-ACLY-WT, Myc-ACLY-S455A, or Myc-ACLY-455D constructs. Bar graphs represent mean ± SEM. ***p < 0.001. a represents ***p < 0.001 vs control (EV), and b represents ***p < 0.001 vs *Rictor-iKO*^*PBA*^ (EV). Statistical significance was calculated by using two-way ANOVA with Sidak’s test multiple comparisons (**b**, **e**, **f**).

To test whether a defect in ACLY phosphorylation explains why mTORC2-deficient brown preadipocytes cannot differentiate, we first determined whether ACLY itself is required for differentiation. To this end, we generated *Acly-iKO*^*PBA*s^ and subjected them to a differentiation assay [Fig. 3a–c]. Indeed, *Acly-iKO*^*PBAs*^ are incapable of differentiating as indicated by their inability to induce *Pparγ2* mRNA or protein [Fig. 3b, Supplementary Fig. 3A] and synthesize lipid droplets [Fig. 3c] in a differentiation assay. Similar results were obtained with two of the three independent short hairpin RNAs (shRNAs) designed to target ACLY [Supplementary Fig. 3B, C]. This confirms that mTORC2 and ACLY are both necessary for brown adipocyte differentiation.

To determine whether mTORC2 and ACLY might function in the same pathway, we asked whether rescuing ACLY^S455^ phosphorylation in *Rictor-iKO*^*PBAs*^ is sufficient to restore their ability to differentiate. As shown previously^11^, *Rictor-iKO*^*PBA*s^ cannot induce *Pparγ* 2 [Fig. 3b] or synthesize lipid droplets [Fig. 3c] in a differentiation assay. Next, we expressed empty vector (EV) or recombinant Myc-ACLY-WT, Myc-ACLY-S455A, or Myc-ACLY-S455D in *Rictor-iKO*^*PBAs*^ [Supplementary Fig. 3D] and differentiated them. Expressing Myc-ACLY-S455D in *Rictor-iKO*^*PBAs*^ restores lipid droplet synthesis (to 108.0% of control) [Fig. 3d] and the expression of *Pparγ2* mRNA (to 74.1% of control) and protein [Fig. 3e, Supplementary Fig. 3E]. ACLY-S455D also restores total acetyl-CoA levels in *Rictor-iKO*^*PBAs*^ to higher than control levels (150.2%) [Fig. 3f]. Thus rescuing ACLY S455 phosphorylation is sufficient to rescue differentiation and acetyl-CoA synthesis in *Rictor-iKO*^*PBAs*^. In contrast, neither vector control nor ACLY-S455A restores lipid droplet synthesis, *Pparγ2* expression, or acetyl-CoA levels [Fig. 3d–f]. Interestingly, overexpressing ACLY-WT slightly rescues lipid droplet synthesis (to 19.2% of control) [Fig. 3d] and *Pparγ2* expression (to 39.0% of control) [Fig. 3e]. However, ACLY-WT does not increase total cellular acetyl-CoA levels [Fig. 3f], consistent with S455 phosphorylation being required for maximal ACLY activity. These data support a model in which ACLY is a critical mTORC2 effector during brown adipogenesis.

### ACLY stimulation requires AKT HM phosphorylation

**Fig. 4:**
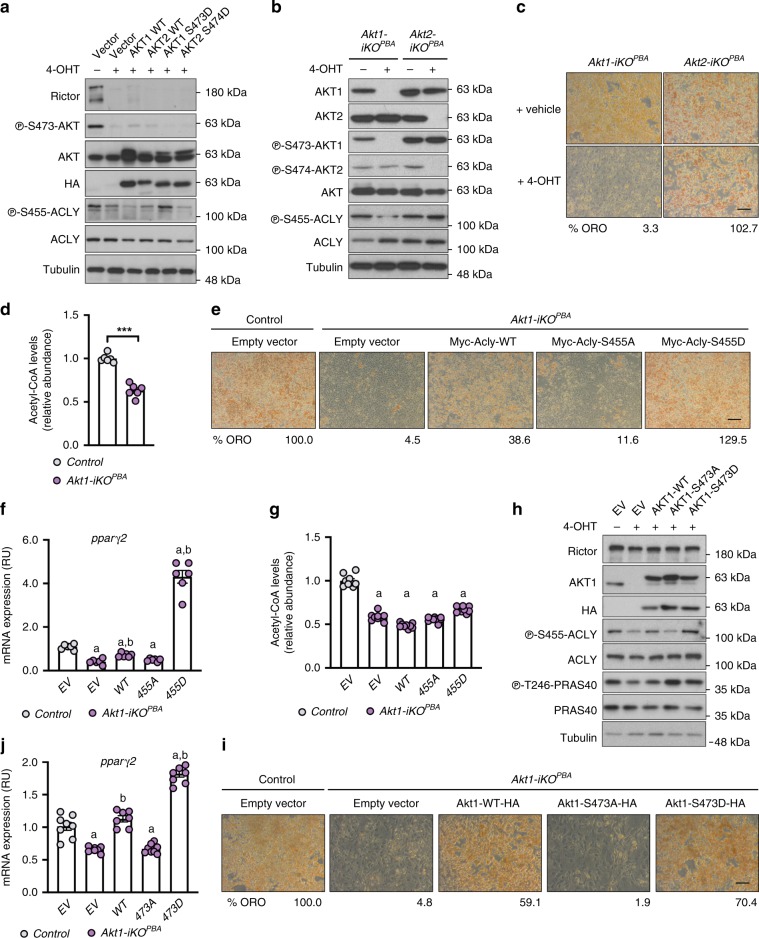
AKT1 HM phosphorylation is necessary for ACLY stimulation and differentiation, while ACLY-S455D is sufficient to rescue acetyl-CoA levels and differentiation upon *Rictor* loss. **a** *Rictor-iKO*^*PBAs*^ cells were stably transfected with the indicated HA-tagged AKT constructs, which were tested for their ability to rescue ACLY-S455 phosphorylation by western blot. **b**, **c** Western blot (**b**) and ORO (**c**) of *Akt1-iKO*^*PBAs*^ and *Akt2-iKO*^*PBAs*^ cells. Scale bar represents 50 μm. **d** Direct acetyl-CoA measurements of *Akt1-iKO*^*PBAs*^ cells (*n* = 6 per group). **e**, **f** Representative differentiation rescue experiments showing ORO staining (**e**) and *pparγ* expression (*n* = 6 per group) (**f**) in *Akt1-iKO* cells stably expressing empty (EV), Myc-ACLY-WT, Myc-ACLY-S455A, or Myc-ACLY-455D constructs. Scale bar represents 50 μm. **g** Direct acetyl-CoA measurements of *Akt1-iKO*^*PBA*^ cells (*n* = 9) stably transfected with the indicated Myc-tagged ACLY constructs. **h**–**j** Western blot (**h**), *pparγ* expression (*n* = 7–8 per group) (**i**), and ORO staining (**j**) of *Akt1-iKO*^*PBA*^ stably expressing empty (EV), HA-AKT1-WT, HA-AKT1-S473A, or HA-AKT1-473D constructs. Scale bar represents 50 μm. Bar graphs represent mean ± SEM. ***p < 0.001, a represents ***p < 0.001 vs control (EV), and b represents ***p < 0.001 vs *Akt1-iKO*^*PBA*^ (EV). Statistical significance was calculated by using two-tailed unpaired Student’s *t* test (**d**) or two-way ANOVA with Sidak’s test multiple comparisons (**f**, **g**, **i**).

That pACLY^S455^ is partially sensitive to mTORC2 loss while direct mTORC2 sites at pAKT1^S473^ and pAKT2^S474^ are highly sensitive is consistent with mTORC2 indirectly stimulating ACLY. Moreover, expressing recombinant AKT1-S473D in *Rictor-iKO* brown preadipocytes mostly rescues their ability to differentiate^11^. These data suggest that AKT1 may be the intermediary between mTORC2 and ACLY during brown adipogenesis, rather than mTORC2 directly phosphorylating ACLY^19^. Indeed, recombinant AKT1-S473D but not AKT1, AKT1-S473A, AKT2, or AKT2-S474D rescues ACLY^S455^ phosphorylation [Fig. 4a], consistent with ACLY being an mTORC2-dependent AKT1 substrate in brown preadipocytes.

To further test this hypothesis, we generated *Akt1-iKO*^*BPAs*^ and *Akt2-iKO*^*BPAs*^ for comparison in a differentiation assay [Fig. 4b]. Like *Rictor-iKO*^*PBAs*^, only the *Akt1-iKO*^*BPAs*^ are incapable of synthesizing lipid droplets in a differentiation assay [Fig. 4c], which is in agreement with previous studies^11,27^. Consistently, *Akt1-iKO*^*BPAs*^ have reduced pACLY^S455^ phosphorylation [Fig. 4b] and reduced acetyl-CoA levels (by 41.2%) [Fig. 4d]. Moreover, the ability of *Akt1-iKO*^*BPAs*^ to differentiate is rescued with Myc-ACLY-S455D (and to a lesser extent with Myc-ACLY-WT) [Fig. 4e, f]. Notably, Myc-ACLY-S455D does not rescue total acetyl-CoA levels [Fig. 4g]. This is consistent with total AKT1 deletion having broader effects on glucose-dependent acetyl-CoA synthesis than losing only HM phosphorylation. Further consistent with this notion, glucose uptake is reduced by 37.8% in *Akt1-iKO*^*BPAs*^ compared to only a 10.89% in *Rictor-iKO*^*PBAs*^ [Supplementary Fig. 4A]. These data support a model in which mTORC2-dependent AKT1 HM phosphorylation stimulates ACLY to promote brown adipocyte differentiation.

If our hypothesis is correct, then we reasoned that brown preadipocytes lacking only the AKT1 HM phosphorylation site, in contrast to total *Akt1* deletion, should phenocopy *Rictor* loss with respect to ACLY phosphorylation and differentiation. To test this, we generated *Akt1-iKO*^*PBA*s^ expressing either EV or recombinant HA-AKT1-WT, HA-AKT1-S473A, or HA-AKT1-S473D. In the *Akt1-iKO*^*PBAs*^-expressing EV, pACLY^S455^ and pPRAS40^T246^ are both attenuated, while in the HA-AKT1-WT- and HA-AKT1-S473D-expressing cells, both sites are phosphorylated as expected [Fig. 4h]. Importantly, pACLY^S455^, but not pPRAS40^T246^, decreases in the HA-AKT1-S473A-expressing cells [Fig. 4h]. Moreover, while HA-AKT1-WT- and HA-AKT1-S473D-expressing cells can differentiate and restore the acetyl-CoA levels, HA-AKT1-S473A cells are incapable [Fig. 4i, j, Supplementary Fig. 3F]. We obtained similar results with *Akt1,2,3* triple KO precursors [Supplementary Fig. 3G, H]. These data confirm that mTORC2-dependent AKT1 HM phosphorylation stimulates ACLY and brown preadipocyte differentiation.

### A glucose uptake deficiency is not limiting upon mTORC2 loss

Because adipocyte mTORC2 loss impairs glucose uptake in vivo^12–14,28^, we had considered the possibility that a glucose uptake deficiency might be preventing *Rictor-iKO*^*PBA*s^ from differentiating. This seemed unlikely as *Rictor-iKO*^*PBAs*^ have a relatively minor defect in glucose uptake [Supplementary Fig. 4A], and moreover, intracellular and G6P levels are normal [Fig. 2c and Supplementary Data 2]. Nevertheless, we tested whether overexpressing the facilitative glucose transporter Glut1 (the main glucose transporter in preadipocytes) in *Rictor-iKO*^*PBAs*^ can rescue differentiation. Despite Glut1 overexpression more than doubling glucose uptake into *Rictor-iKO*^*PBAs*^ and to levels equivalent with Glut1-overexpressing control cells, this fails to rescue differentiation [Supplementary Fig. 4B–D]. Similarly, overexpressing WT (HK-WT) or hyperactive hexokinase-2 (HK-T473D), which traps glucose inside the cell by phosphorylating the glucose 6 carbon and has previously been linked to mTORC2^29^, also fails to rescue differentiation [Supplementary Fig. 4E, F]. Thus neither a glucose uptake nor a trapping defect is a major limiting factor for the differentiation of *Rictor-iKO*^*BPA*s^.

### mTORC2 regulates histone acetylation during differentiation

Overexpressing recombinant PPARγ2 in *Rictor-iKO* brown preadipocytes rescues differentiation; however, supplementing rosiglitizone (a synthetic PPARγ ligand) cannot^11^, suggesting that the mTORC2/AKT1/ACLY pathway likely regulates an early transcriptional initiation event rather than a defect in endogenous ligand synthesis and/or amplification. Given that ACLY also generates acetyl-CoA necessary for histone acetylation and *Glut4* expression during white adipocyte differentiation^30,31^, we asked whether mTORC2 might facilitate epigenetic programming during brown adipocyte differentiation. Notably, total H3 acetylation decreases in undifferentiated *Rictor-iKO*^*PBAs*^ [Supplementary Fig. 5A]. At differentiating day 2, just before *Pparγ*2 is initially induced, analysis of specific acetylation marks also reveals a marked decrease in H3K27Ac (a key marker of active enhancers during adipogenesis^32^) in *Rictor-iKO*^*PBA*^, *Akt1-iKO*^*PBA*^, and *Acly-iKO*^*PBA*^ cell lines [Supplementary Fig. 5B–D]. Furthermore, H3K27 acetylation in the promoter of *Pparγ*2 and CD36 (a *Pparγ* target gene) greatly decreases in *Rictor-iKO*^*PBAs*^ [Supplementary Fig. 5E]. Finally, expressing recombinant Myc-ACLY-S455D in *Rictor-iKO*^*PBAs*^, but not EV, Myc-ACLY-WT, or Myc-ACLY-S455A, rescues H3K27Ac [Supplementary Fig. 5F]. Thus stimulating ACLY-dependent histone acetylation during brown adipogenesis is an additional mTORC2 function in brown adipocytes.

### mTORC2 regulates acetyl-CoA synthesis from acetate

**Fig. 5:**
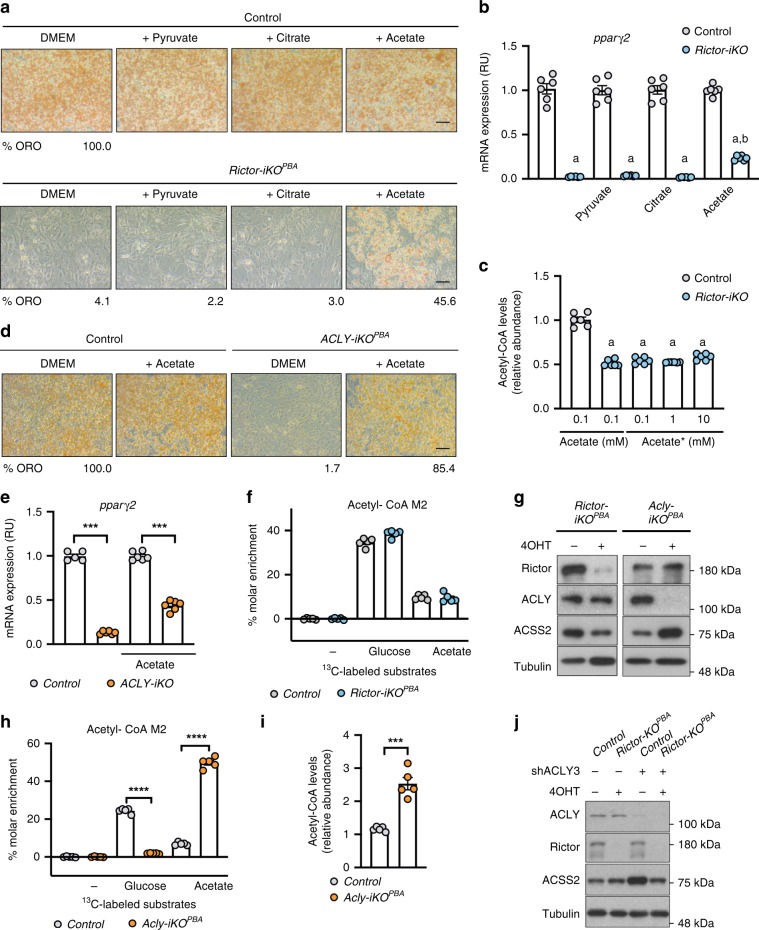
mTORC2 also regulates acetyl-CoA synthesis from acetate. **a**, **b** Isogenic control and *Rictor-iKO* brown preadipocytes were supplemented with the indicated metabolites during differentiation and Oil Red O (ORO) staining of lipids (**a**) and *pparγ2* mRNA expression (*n* = 6 per group) (**b**) were quantified as indicators of differentiation efficiency. Percentage of ORO is relative to control in DMEM. Scale bar represents 50 μm. **c** Direct acetyl-CoA levels in control and *Rictor-iKO*^*PBA*^ cells (*n* = 6 per group) supplemented with unlabeled acetate (0.1 mM) or increasing [1,2-^13^C] acetate concentration. **d**, **e** *Acly-iKO*^*PBA*^ cells were differentiated with or without acetate supplementation followed by Oil Red O (ORO) staining (**d**) and *pparγ2* mRNA expression (*n* = 6 per group) (**e**) analysis. Scale bar represents 50 μm. **f** M2 isotopic tracer labeling of total acetyl-CoA levels in *Rictor-iKO*^*PBA*^ cells (*n* = 5 per group) that were serum deprived for 12 h and then incubated for 1 h with fresh glucose-free DMEM supplemented with 5 mM of labeled [U-^13^C] glucose and 100 μM of unlabeled acetate or unlabeled glucose and 100 μM of [1,2-^13^C] acetate, at 37 °C for 1 h. **g** Western blot showing total ACSS2 protein expression in *Rictor-iKO*^*PBA*^ and *Acly-iKO*^*PBA*^ cells. **h** M2 isotopic tracer labeling of total acetyl-CoA levels in *Acly-iKO*^*PBA*^ cells (*n* = 5) as described in **f**. **i** Direct acetyl-CoA levels in control and *Acly-iKO*^*PBA*^ cells (*n* = 5 per group). **j** Control and *Rictor-iKO* cells were stably transfected with control (scrambled-shRNA) or an shRNA targeting ACLY. Knockdown efficiency and effects on ACSS2 expression were confirmed by western blot. Bar graphs represent mean ± SEM. ***p < 0.001, ****p < 0.0001, a represents ***p < 0.001 vs control (EV), and b represents ***p < 0.001 vs *Rictor-iKO*^*PBA*^ (EV) cells. Statistical significance was calculated by using two-way ANOVA with Sidak’s test multiple comparisons (**b**, **c**, **e**, **f**, **h**) or two-tailed unpaired Student’s *t* test (**i**).

While glucose fuels acetyl-CoA synthesis through ACLY, acetyl-CoA can also be generated from acetate through ACSS2^33^. Therefore, we asked whether the ability of *Rictor-iKO*^*PBAs*^ to differentiate might also be rescued by supplementing high levels of exogenous acetate into the culture medium. We compared this by adding excess exogenous citrate or pyruvate, which are acetyl-CoA precursors in the glucose pathway. Neither citrate nor pyruvate supplementation rescues differentiation [Fig. 5a, b]. However, supplementing acetate partially rescues lipid droplet synthesis (to 45.57% of control) and *Pparγ2* expression (to 23.47% of control) as well as protein expression without restoring acetyl-CoA level [Fig. 5a–c, Supplementary Fig. 5G]. Interestingly, performing the acetate supplementation experiment with *Acly-iKO*^*PBAs*^ indicates that acetate is nearly twice as effective at restoring lipid droplet synthesis and *Pparγ* expression (to 85.4% and 44.0% of control levels, respectively) vs *Rictor-iKO*^*PBAs*^ [Fig. 5d, e, Supplementary Fig. 5H]. That *Rictor-*^*iKOPBA*s^ are less efficient than *Acly-iKO*^*PBAs*^ at using acetate, combined with the fact that acetate supplementation did not restore acetyl-CoA levels, suggested the intriguing possibility that mTORC2 might also regulate acetate-derived acetyl-CoA synthesis.

To test whether mTORC2 regulates acetyl-CoA synthesis from acetate, we performed tracer experiments by culturing *Rictor-iKO*^*PBAs*^ in the presence of [U-^13^C]-glucose (10 mM) and physiological levels of unlabeled acetate (100 μM) or physiological levels of [1,2-^13^C]-acetate (100 μM) and unlabeled glucose (10 mM) for 1 h prior to measuring acetyl-CoA. Compared to their isogenic controls, the percentage of labeling of acetyl-CoA from both glucose and acetate is unchanged in *Rictor-iKO*^*PBA*s^ [Fig. 5f]. As total acetyl-CoA levels are halved in these cells [Fig. 2e], this suggests that *Rictor* loss may reduce the ability of cells to use either glucose or acetate to label acetyl-CoA by roughly equivalent proportions.

In MEFs, deleting *Acly* upregulates *acss2*/ACSS2, thereby enabling cells to engage compensatory acetate metabolism to generate acetyl-CoA^34^. We reasoned that a difference in ACSS2 expression could explain why *Rictor-iKO*^*PBA*s^ use acetate less effectively than *Acly-iKO*^*PBAs*^. Like in MEFs, *Acly-iKO*^*PBAs*^ induce compensatory ACSS2 expression [Fig. 5g]. Glucose and acetate tracer analysis also shows that *Acly-iKO*^*PBAs*^ switch from using glucose to acetate for acetyl-CoA synthesis [Fig. 5h], increasing total cellular acetyl-CoA above normal levels (by 118.14%) [Fig. 5i], also like in MEFs. However, this alternative pool of acetyl-CoA is reportedly not efficiently used for histone acetylation unless high levels of exogenous acetate are supplemented likely explaining why *Acly-iKO*^*PBAs*^ cannot differentiate at physiological acetate levels [Fig. 5d–e]^30^. Consistently, *Acly-iKO*^*PBAs*^ have defects in H3K9 and H3K14 acetylation in addition to H3K27 acetylation that are all rescued with high levels of exogenous acetate [Supplementary Fig. 5D].

In contrast, despite *Rictor-iKO*^*PBAs*^ having nearly a 50% drop in total acetyl-CoA levels, they fail to trigger compensatory ACSS2 induction; in fact, ACSS2 levels are reduced in *Rictor-iKO*^*PBA*^ cells [Fig. 5g]. This is consistent with *Rictor*-deficient cells having reduced capacity to metabolize acetate. Moreover, while *Rictor-iKO*^*PBAs*^ and *Acly-iKO*^*PBAs*^ overlap in having both H3K14Ac and H3K27Ac deficiencies, only H3K27Ac is rescued with high acetate supplementation in *Rictor*-deficient cells [Supplementary Fig. 5B, D]. Finally, we show that compensatory ACSS2 upregulation triggered by ACLY loss is blocked when *Rictor* is simultaneously knocked out [Fig. 5j] indicating the glucose-to-acetate metabolic switch triggered upon ACLY deletion also requires mTORC2. Collectively, these data reveal an additional role for mTORC2 in promoting acetyl-CoA synthesis from acetate at least in part by controlling ACSS2 expression.

### ACLY is a key mTORC2 effector in mature brown adipocytes

Our prior in vivo observations show that chronic mTORC2 loss (caused by conditionally deleting *Rictor* in mature brown or white adipocytes with congenitally expressed Cre drivers) attenuates ACLY, ACC, and FASN mRNA and protein expression^11–13^. In contrast, more acutely deleting *Rictor* in precursor brown adipocytes by inducible KO reduces gluco-lipogenic flux but did not impair DNL enzyme expression [Fig. 2f, Supplementary Fig. 1B, C]. This led us to consider whether the decrease in *Acly*, *Acc*, and *Fasn* gene expression that we observe in mature brown adipocytes may be a secondary effect of impaired ACLY function that occurs with prolonged *Rictor* loss.

**Fig. 6:**
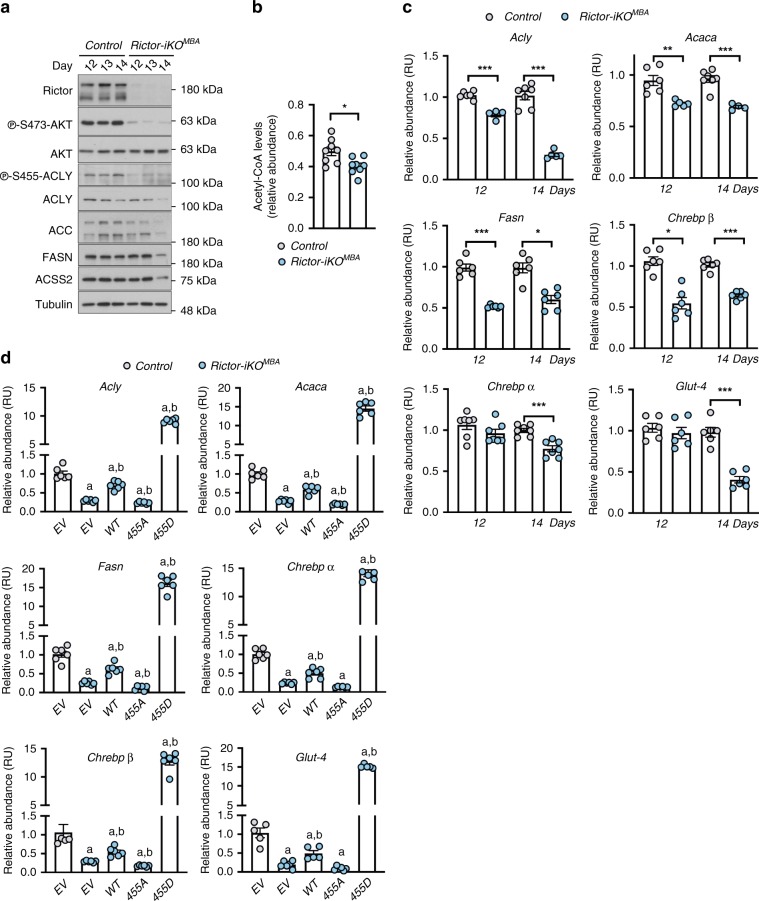
mTORC2 drives lipogenic and *Glut4* gene expression through ACLY in mature brown adipocytes. **a** Time-course western blots showing the indicated phospho-specific and total proteins in *Rictor-iKO*^*MBA*^ or *Acly-iKO*^*MBA*^ cells following acute and prolonged protein loss. **b** Direct acetyl-CoA measurements in *Rictor-iKO*^*MBA*^ cells following acute *Rictor* loss (day 12) (*n* = 8 per group). **c** qRT-PCR analysis of the indicated genes following acute (day 12) and prolonged (day 14) *Rictor* loss (*n* = 6 per group). *Rictor* deletion was induced at day 8 during differentiation. **d** qRT-PCR analysis of the indicated genes *Rictor-iKO*^*MBA*^ (*n* = 6 per group) stably expressing empty (EV), Myc-ACLY-WT, Myc-ACLY-S455A, or Myc-ACLY-455D constructs. Bar graphs represent mean ± SEM. *p < 0.05, **p < 0.01, ***p < 0.001, a represents ***p < 0.001 vs control (EV), and b represents ***p < 0.001 vs *Rictor-iKO*^*MBA*^ (EV) cells. Statistical significance was calculated by using two*-*tailed unpaired Student’s *t* test (**b**) or two-way ANOVA with Sidak’s test multiple comparisons (**c**, **d**).

To investigate the temporal effect of acute vs prolonged *Rictor* loss on gluco-lipogenic gene expression, we performed a time-course analysis of ACLY phosphorylation and gene expression following tamoxifen-induced *Rictor* deletion in mature brown adipocytes (hereafter *Rictor-iKO*^*MBAs*^). Briefly, *UBC-Cre;Rictor*^*L/L*^ brown adipocyte precursors were treated with 4-hydroxy-tamoxifen (4-OHT) at day 8 of differentiation, after *Pparγ2* induction, to generate *Rictor-iKO*^*MBAs*^ [Supplementary Fig. 6A, B]. Shortly after 4-OHT treatment (corresponding to differentiation day 12), mature brown adipocytes are depleted of *Rictor* and have attenuated pAKT^S473^ and pACLY^S455^ [Fig. 6a]. This coincides with a 19% reduction in acetyl-CoA levels [Fig. 6b] and reduced H3K27Ac [Supplementary Fig. 5I]. At this time point, ACLY, ACC, and FASN protein levels are largely unaffected [Fig. 6a], while *Acly*, *Acc*, and *Fasn* mRNA show a 28.0%, 28.8% and 48.3% decrease, respectively [Fig. 6c]. We also observe a 45.3% decrease in *Chrebpβ* mRNA expression, while *Chrebpα* and *Glut4* expression are normal [Fig. 6c]. In contrast, more prolonged *Rictor* loss (differentiation day 14) results in a 62.2% decrease in *Glut4* mRNA expression and depletion of ACLY, ACC, and FASN protein [Fig. 6a, c]. In addition, ACSS2 levels decrease at day 14 consistent with mTORC2 also regulating its expression in vitro in mature brown adipocytes [Fig. 6a]. These data are consistent with *Rictor* loss acutely impairing ACLY activity, histone acetylation, and lipogenic gene expression, which precedes downregulation of DNL proteins and glucose transporter expression.

Next, we asked whether expressing the recombinant Myc-ACLY-S455D in *Rictor-iKO*^*MBAs*^ could rescue gluco-lipogenic gene expression. Indeed, overexpressing ACLY-S455D rescues *Acly*, *Acc*, *Fasn*, *Chrebpβ*, and *Glut4* expression and to higher than control levels [Fig. 6d]. Interestingly, overexpressing WT-ACLY also moderately rescues gene expression [Fig. 6d], while in contrast, vector alone has little effect and ACLY-S455A has a mild negative effect [Fig. 6d]. These data are consistent with ACLY being a critical mediator between mTORC2, ChREBP, and gluco-lipogenic gene expression in mature brown adipocytes.

### mTORC2 and ACLY loss affect the DNL pathway in vivo

**Fig. 7:**
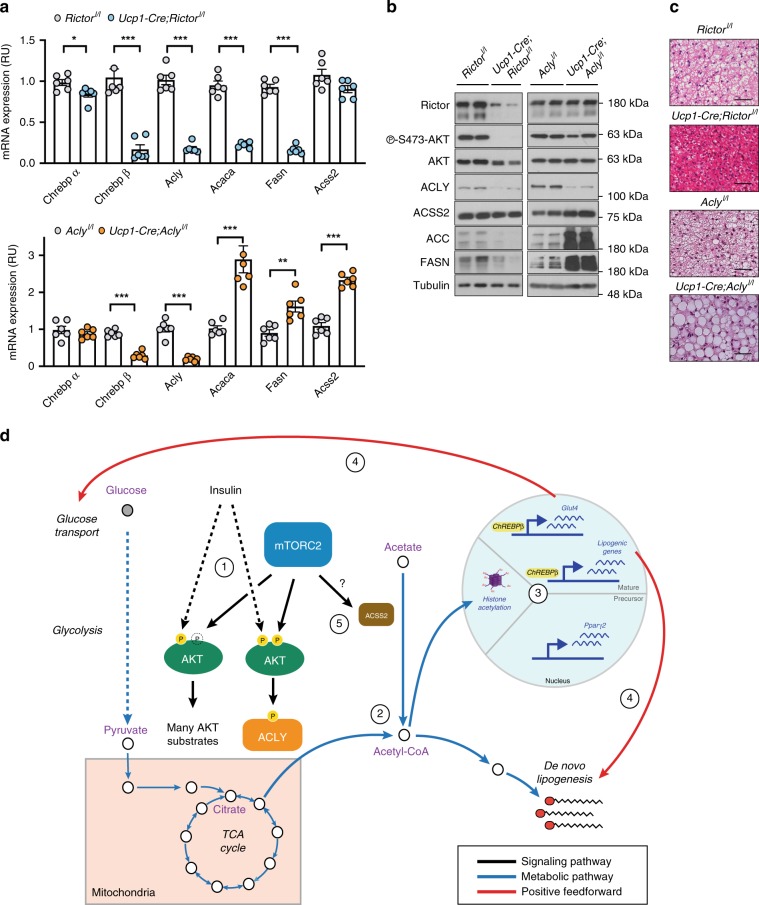
In vivo, mTORC2 and ACLY both promote ChREBPβ expression, but their loss has opposite effects on ACC and FASN expression that correlates with differential ACSS2 regulation. **a** qRT-PCR analysis of the indicated genes in brown fat tissue isolated from control (*Rictor*^*l/l*^ *and Acly*^*l/l*^) or *UCP1-Cre-Rictor*^*l/l*^ and *UCP1-Cre-Acly*^*l/l*^ mice (*n* = 6 per group). Bar graphs represent mean ± SEM. *p < 0.05, **p < 0.01, and ***p < 0.001. Statistical significance was calculated by using two-way ANOVA with Sidak’s test multiple comparisons (**a**). **b** Corresponding western blots for **a**. Black arrows indicate the ACC1 and ACC2 isoforms. **c** Hematoxylin and eosin stains showing brown fat morphology for the indicated genotypes. Scale bar represents 200 μm. **d** Our results support the following model of mTORC2 action in brown adipocytes: (1) mTORC2-dependent AKT phosphorylation is distinctly important for ACLY phosphorylation, while for many other AKT substrates, mTORC2 may normally facilitate their phosphorylation, but it is dispensable (indicated by “P” within an unshaded broken circle). This is indicated by the fact that phosphorylation of many AKT substrates is unimpaired by *Rictor* deletion while ACLY phosphorylation is reduced. (2) mTORC2/AKT-dependent ACLY phosphorylation promotes acetyl-CoA synthesis and primes de novo lipogenesis downstream of glucose uptake and glycolysis. (3) The increased flux to acetyl-CoA additionally stimulates histone acetylation. In brown adipocyte precursors, this coincides with *pparγ* induction to drive differentiation; in mature brown adipocytes, this coincides with ChREBPβ activity to increase expression of gluco-lipogenic genes. (4) Gluco-lipogenic gene expression then provides a positive feedback effect on glucose transport and de novo lipogenesis. (5) mTORC2 may also stimulate acetate metabolism to acetyl-CoA by regulating the expression and/or activity of ACSS2. Notably, from these data we cannot distinguish between cytoplasmic and nuclear pools of acetyl-CoA.

Finally, we explored the in vivo physiological relevance of these findings by comparing the effects of deleting *Rictor*^*l/l*^ or *Acly*^*l/l*^ alleles in BAT with *Ucp1-Cre*. As we recently reported^13^, the BAT of *Ucp1-Cre;Rictor*^*l/l*^ mice have dramatically attenuated *Chrebpβ*, *Acly*, *Acaca*, and *Fasn* mRNA [Fig. 7a] and protein expression [Fig. 7b] and small lipid droplets [Fig. 7c]. *Rictor*-deficient BAT also has reduced H3K27 acetylation [Supplementary Fig. 5J]. However, despite the low *Acly* expression, which is comparable to deleting *Acly* itself, *Acss2*/ACSS2 levels remain largely unchanged [Fig. 7a, b]. This is consistent with mTORC2 being necessary to invoke the ACSS2-driven glucose-to-acetate metabolic switch. In contrast *Ucp1-Cre;Acly*^*l/l*^ mice expresses higher levels of *Acaca*/ACC and *Fasn*/FASN mRNA and protein [Fig. 7a, b] and have larger lipid droplets [Fig. 7c]. Increased BAT lipid droplet size is also evident in the BAT of *Adiponectin-Cre;Acly*^*l/l*^ mice [Supplementary Fig. 7]. This phenotypic divergence correlates with *Acss2*/ACSS2 expression, which is induced in *Acly* but not in *Rictor*-deficient BAT [Fig. 7a, b]. Interestingly, *Chrebpβ* expression decreases in the BAT of both models [Fig. 7a] indicating that *Chrebpβ* may not be driving *Acss2* expression in *Acly*-deficient BAT. This is also consistent with ChREBP functioning downstream of both mTORC2 and ACLY^12,35^.

Thus, while the effects of deleting *Rictor* or *Acly* during brown adipocyte differentiation are largely overlapping, their loss in mature brown adipocytes has opposite effects on ACSS2 expression, downstream DNL pathway expression, and lipid droplet content. This is despite the fact that deleting *Rictor* or *Acly* decreases *Acly*/ACLY expression to nearly the same extent. These data are consistent with mTORC2 additionally regulating ACSS2 in a physiologically relevant context and supports an overarching model in which brown adipocyte mTORC2 regulates glucose- and acetate-derived acetyl-CoA synthesis [Fig. 7d].

## Discussion

Prior studies implicate adipocyte mTORC2 in controlling insulin-stimulated glucose uptake and DNL (gluco-lipogenesis). However, the mechanism has been enigmatic because most AKT substrates examined appear to be phosphorylated normally when *Rictor* is deleted^11–13^. Solving this mystery is important because a similar state of adipose tissue “selective insulin resistance” in which insulin-stimulated glucose uptake, but not AKT activity per se, is defective, occurs in several models of adipocyte insulin resistance^36,37^. Moreover, DNL in human BAT and WAT positively correlates with thermogenesis and insulin sensitivity, respectively^15,38–40^. In this study, we uncover a previously unappreciated selectivity in mTORC2-dependent AKT signaling toward ACLY that provides a plausible mechanistic underpinning for why mTORC2 loss impairs brown adipocyte glucose-dependent lipogenesis and differentiation.

One limitation of this study is that it does not address why ACLY^S455^ is different from other AKT substrates. Although our inducible KO system results in rapid loss of *Rictor* and AKT HM phosphorylation, it remains possible that a compensatory mechanism stabilizes T308 phosphorylation maintaining AKT activity toward most substrates. A selective mTORC2 inhibitor (i.e., which does not inhibit mTORC1) would be valuable in determining the acute effects of mTORC2; however, such a molecule is not yet available. Nevertheless, why would ACLY phosphorylation differ from other AKT substrates in its mTORC2 dependency? One possibility is that intrinsic differences in the ACLY S455 motif may demand higher AKT activity, or a different biochemical conformation dependent on HM phosphorylation, to be efficiently phosphorylated^41^^,^^42^. Aligning mTORC2 dependent and independent phosphosites identified in this study to the AKT consensus motif indicates that more dependent sites are less conserved [Supplementary Fig. 2D]. Alternatively, the mTORC2 dependency of ACLY phosphorylation could relate to its localization and/or temporal dynamics of phosphorylation. For example, ACLY phosphorylation may occur distal to the plasma membrane and/or membrane PIP3 and therefore may require HM phosphorylation to recruit PDK1, which is how other AGC family kinases lacking PH domains are regulated^4^. Indeed, evidence suggests that AKT is regulated by both PH-domain-dependent and -independent mechanisms in cancer cells^43^. Proteomics and western blotting data also indicate that mTORC2 loss decreases ACLY phosphorylation during serum deprivation suggesting that mTORC2 might regulate acetyl-CoA synthesis in multiple nutritional states. Another possibility is that mTORC2 may suppress an ACLY phosphatase.

Two studies comparing temporal dynamics of the insulin-stimulated phosphoproteome and metabolome in 3T3-L1 adipocytes show that total citrate levels acutely decrease, and acetyl-CoA levels increase, roughly when ACLY S455 phosphorylation is stimulated and that this occurs prior to maximum glucose transport^44,45^. This suggests that mTORC2-dependent ACLY stimulation may be an early insulin action that mobilizes metabolites downstream of glucose transport, glycolysis, and the TCA cycle to both relieve citrate’s allosteric inhibitory effect on PFK1 and prime gluco-lipogenesis. Data from this study further suggest that mTORC2-dependent ACLY phosphorylation additionally creates an overflow of acetyl-CoA that is siphoned toward histone acetylation, thereby providing an insulin-coupled secondary epigenetic signal that is a direct readout of systemic nutrient (glucose) availability. We suggest a model that, in brown adipocyte precursors, this pathway provides a license for differentiation, while in mature brown adipocytes, it potentiates glucose uptake, glycolysis, and lipid synthesis by upregulating and/or maintaining ChREBP activity and the gluco-lipogenic transcriptional program [Fig. 7d]. Thus mTORC2-simulated acetyl-CoA synthesis may provide a key missing link between systemic glucose availability and setting the genetic program that determines the glucose- and lipid-handling capacity of brown adipocytes.

mTORC2 also promotes *Chrebpβ* expression, which drives gluco-lipogenic gene expression including that of *Acly*^12,13^. Interestingly, *Chrebpβ* expression is also ACLY dependent in white adipocytes^31^ consistent with ChREBPα/*β* functioning downstream of mTORC2/ACLY in a positive feedback loop. Thus ChREBP could also be the key target of mTORC2/ACLY in gluco-lipogenic gene regulation. In fact, hepatocyte ChREBP transcriptional activity is reportedly regulated by acetylation and *O*-GlcNacylation, which both require acetyl-CoA precursors^46,47^. The most likely scenario is that histone acetylation and ChREBP activity are interconnected, and further resolving how ChREBP is regulated by mTORC2 and ACLY will be important. The mTORC2/ACLY pathway might additionally regulate other posttranslational modifications or allosteric regulatory roles linked to DNL metabolites, such as palmitoylation, which is implicated in Glut4 trafficking^48,49^. The potential role of DNL as a source of signaling metabolites in adipocytes has been underappreciated, but it is tempting to speculate that defects in their second messenger functions could be key steps in the early onset of insulin resistance.

That mTORC2 additionally regulates acetyl-CoA synthesis from acetate indicates a broader role of mTORC2 in controlling nuclear–cytoplasmic acetyl-CoA synthesis. The role of acetate as a metabolic substrate in adipocytes is not well understood. However, the differential effects on ACSS2 expression caused by *Rictor* loss vs *Acly* loss could explain why *Adiponectin-Cre;Rictor*^*l/l*^ mice have a more severe metabolic phenotype compared to *Adiponectin-Cre;Acly*^*l/l*^ mice^12,34^. In conclusion, the identification of ACLY and ACSS2 as key targets of mTORC2 action in brown adipocytes fills an important gap in our understanding of this more mysterious mTOR complex. These findings may be relevant to understanding mTORC2 action in other tissues and in the pathogenesis of type-2 diabetes.

## Methods

### Mice

C57BL/6J mice (JAX stock 000664) were obtained from Jackson Laboratory. *Ucp1-Cre* (JAX stock 024670) and *Adiponectin-Cre* (JAX stock 010803) were kindly provided by Dr. Evan Rosen. *Akt1*^*l/l*^, *Akt2*^*l/l*^, and *Akt3*-KO mice were kindly provided by Dr. Birnbaum MJ. Ubc-Cre^ERT2^; *Rictor*^*l/l*^ mice and *Acly*^*l/l*^ mice were generated by crossing UBC-Cre^ERT2^ with *Rictor*^*l/l*^ or *Acly*^*l/l*^ mice^11,34^. UBC-Cre^ERT2^; *Akt1*^*l/l*^*, Akt2*^*l/l*^*, Akt3-KO* were generated by crossing UBC-Cre^ERT2^; *Akt1*^*l/l*^ with UBC-Cre^ERT2^; *Akt2*^*l/l*^ until UBC-Cre^ERT2^; *Akt1*^*l/l*^*, Akt2*^*l/l*^ was obtained and then once more to Akt3-KO mice until we obtained the final Ubc-Cre^ERT2^; *Akt1*^*l/l*^*, Akt2*^*l/l*^*, Akt3-KO* genotype. Mice were housed in the UMMS Animal Medicine facilities in a room set at 22 °C in 45% humidity under daily 12 light/dark cycles. For all in vivo studies, we used 8-week-old *Ucp1-Cre-Rictor*^*l/l*^ and *Ucp1-Cre-Acly*^*l/l*^ mice. We have complied with all relevant ethical regulations for animal testing and research, and all animal studies have been approved by the Umass Medical School Committee on Animal Care (IACUC).

### Cultured cells

Brown preadipocytes were isolated from Ubc-Cre^ERT2^; *Rictor*^*l/l*^, Ubc-Cre^ERT2^; *Acly*^*l/l*^, or Ubc-Cre^ERT2^; *Akt1*^*l/l*^*, Akt2*^*l/l*^*, Akt3*^−/−^ P1 neonates and immortalized with pBabe-SV40 Large T according to a standard protocol^50^. Cells stably expressing recombinant proteins were obtained either by using lentiviral (TRCN 0000055214-17) and Rictor (addgene: P1853/1854) shRNA experiments or retroviral systems (e.g., Glut-1, HK, Akt, and Acly experiments). For brown adipocyte differentiation, cells were seeded and allowed to proliferate to confluence in the presence of differentiation medium that includes 20 mM of insulin and 1 nM of T_3_. At day 4, cells were treated with induction medium containing 20 mM of insulin, 1 nM of T_3_, 0.125 mM of indomethacin, 2 μM/mL dexamethasone, and 0.5 mM 3-isobutyl-1-methylxantine (IBMX) for 2 days. Then the induction medium was replaced with differentiation medium that was changed every 2 days until day 14. Deletion of *Rictor*, *Acly*, *Akt1*, and/or *Akt2* was achieved by treating the cells with two doses of 4-OHT (1 μM) prior to the start of differentiation (to obtain *Rictor-iKO*^*PBA*^ cells) or one dose at day 8 of differentiation (to obtain *Rictor-iKO*^*MBA*^ cells) depending on the experiment. Control cells received an equivalent dose of ethanol (vehicle). To analyze insulin or fetal bovine serum (FBS) signaling, cells were serum deprived for 12 h and then stimulated with insulin (150 nm) or 10% FBS for 15 min. To analyze AKT dependency, cells were treated with or without AKT inhibitor MK2206 (10 μM). For metabolite rescue experiments, pyruvate (2.5 mM), citrate (2.5 mM), or sodium acetate (5 mM) was added in every media change to the corresponding differentiation or induction media. For Myc-ACLY-WT, Myc-ACLY-S455A, and Myc-ACLY-S455D overexpression experiments in *Rictor-iKO*^*MBA*^, cells were infected at day 10 of differentiation and collected at day 14. HEK293E cells were grown in 10% FBS in Dulbecco’s modified Eagle’s medium (DMEM) and infected with pLentiCRISPRv2 (addgene:52961) with a guide targeting the first exon of *Rictor* (CCCGTCAATATGGCGGCGTCGG) and were selected in puromycin for 4 days. *Rictor* KO was validated by western blot. Rictor was re-expressed in these cells and validated by western blot.

### Antibodies and reagents

GLUT-1 (ab652) and H3K27Ac (ab4729) were purchased from Abcam. HK (A0994) and horseradish peroxidase (HRP)-goat anti-Rabbit were purchased from ABclonal. HRP-conjugate anti-mouse (w402b) was purchased from Promega. H2BK (07-373), H4K (06-866), and H3K (06-599) were purchased from Millipore. Flag (F7425) was purchased from Sigma-Aldrich. All other antibodies including Rictor (2140), PRAS40 (2691), AKT (9272), GSK3b (9315), ACC (3676), ACLY (4332), FASN (3180), PPARg (2443), ACSS2 (3658), S6 (2217), S473-AKT (4058), T308-AKT (4056), S455-ACLY (4331), T246-pPRAS40 (2997), HA-tag (2367), S9-GSK3b (9315), S235/236-S6 (4858), H3 (9715), H3K9Ac (9649), H3K14Ac (7627), and a-Tubulin (2125) were purchased from Cell Signaling Technologies. 4-OHT was obtained from Toronto Research Chemicals. D-[U-^14^C]-glucose (NEC042V250UC) was purchased from Perkin Elmer. MK2206 (S1078) was purchased from Selleck Chemicals. T3 (T2877), dexamethasone (D1756), IBMX (I5879), and insulin (I2643) were from Sigma-Aldrich. [U-^13^C] glucose (CLM-1396-1) and [1,2-^13^C] acetate (CLM-440-PK) were purchased from Cambridge Isotope Laboratories, Inc.

### Western blot analysis

Cells were homogenized in a Triton-X lysis buffer containing 150 mM NaCl, 50 mM Tris pH 7.5, 0.5% deoxycholate, 1% Triton X-100, and 0.1% sodium dodecyl sulfate (SDS); a protease inhibitor cocktail (Biotool Cat. Number); and a phosphatase inhibitor cocktail (Sigma Cat. Number P5726). For in vivo analysis, each tissue was collected and frozen down immediately in liquid nitrogen and then stored at −80 °C for subsequent lysis. Tissues were homogenized using a TissueLyser (Qiagen) in RIPA lysis buffer containing 150 mM NaCl, 1% NP-40, 0.5% deoxycholate, 0.1% SDS, and 50 mM Tris, pH 7.5 including a protease (Biotool Cat. Number B14011) and phosphatase inhibitor cocktail (Sigma Cat. Number P5726). An equal amount of total protein was loaded into either 10% acrylamide/bis-acrylamide gels for general analysis or 16% acrylamide/bis-acrylamide gels for histone analysis and transferred to polyvinylidene fluoride membranes. Ponceau stain was performed to check for equal transfer and loading. Membranes were incubated in either 5% milk/phosphate-buffered saline with Tween detergent (PBST) or 5% bovine serum albumin (BSA)/PBST for 1 h and with primary antibodies (1:1000 dilution) in 5% milk/PBST or 5% BSA/PBST overnight, washed 3 times with PBST, and then incubated with the correspondent secondary antibody for 2 h. Tubulin was used as an additional protein level control where indicated. HRP-conjugated secondary antibodies (1:10,000 dilution) were given for 1 h. Western blots were developed by enhanced chemiluminescence (PerkinElmer) and detected by X-ray films.

### Chromatin extraction

Chromatin extraction was performed prior to the western blot analysis^51^. Briefly, cells were collected at day 2 of the differentiation assay, before *Pparγ2* induction, resuspended in lysis buffer (10 mM Hepes pH 7.4, 10 mM KCl, 0.05% NP-40, and a protease (Biotool Cat. Number B14011) and phosphatase inhibitor cocktail (Sigma Cat. Number P5726)) and incubated on ice for 20 min. Then the samples were centrifuged at 16,000 × *g* for 10 min after which the nuclear pellet was resuspended in HCl 0.2 N and incubated on ice for an additional 20 min. Samples were centrifuged at 16,000 × *g* for 10 min, and the supernatant containing the chromatin was neutralized in Tris-HCl pH 8 1 M.

### Glucose uptake

Cells were preincubated for 3 h in a medium without glucose (KRH medium (Gibco plus 0.5% BSA + 2 mM pyruvate). Deoxy-d-glucose 2-[1,2-^3^H(N)] was then added, and incubation was continued for an additional 10 min. The medium was then removed and cells were washed three times with KRH medium to terminate the assay. Cells were then lysed in 1% triton, mixed with scintillation buffer, and the uptake of ^3^H glucose was quantified in counts per minute (cpm) using a scintillation counter. The cpm values were normalized to the protein concentration of each sample.

### Phosphoproteomics

Control and *Rictor-iKO* cells were serum deprived for 12 h (unstimulated samples) and then some were treated with 150 nM insulin for 15 min (stimulated). All cells were washed 3 times with cold PBS, snap-frozen in liquid nitrogen, and thawed in a lysis buffer (8 M urea, 50 mM Tris pH 8.2, 75 mM NaCl, 50 mM β-glycerophosphate, 1 mM sodium orthovanadate, 10 mM sodium pyrophosphate, 50 mM sodium fluoride, and 1× EDTA-free protease inhibitor cocktail (Roche)). Each well of a 6-well plate was scraped in 125 μL of ice-cold lysis buffer, and 2 wells were pooled for each replicate. Lysates were probe-sonicated two times, and clarified by centrifugation at 16,000 × *g*. Protein content was measured by a BCA assay (Pierce). Samples were reduced for 45 min at 55 °C using 5 mM dithiothreitol, alkylated with 15 mM iodoacetamide for 30 min in the dark at room temperature, and quenched with an additional 5 mM of dithiothreitol at room temperature for 15 min. Urea was diluted to 1.5 M using 50 mM pH 8.2 Tris, and protein was digested for 16 h using 5 μg/mL trypsin at 37 °C. Digested peptides were de-salted using 50 mg Sep-Pak cartridges (Waters) and dried by vacuum centrifugation. Phosphopeptides were enriched using PureCube Fe-NTA magnetic beads (Cube Biotech) in a KingFisher magnetic bead processor (Thermo). Dried phosphopeptide samples were prepared for analysis by liquid chromatography coupled to tandem mass spectrometry (LC-MS/MS) by dissolution in 4% formic acid and 3% acetonitrile.

To screen for mTORC2-dependent phosphorylation, we designed a targeted, parallel reaction monitoring (PRM) mass spectrometry method that was informed by manual literature curation and prior preliminary experiments. To design the list, we curated a preliminary list of 228 phosphopeptides representing previously characterized AKT substrates, regulatory phosphorylation sites on AGC kinases, phosphorylation sites on proteins with known regulatory function in insulin signaling and/or brown fat, and phosphorylation sites with potential mTORC2 dependence according to preliminary, unpublished data. According to a previously reported method^52^, we referenced an in-house database of mouse phosphoproteome data gathered from several different tissues using data-dependent acquisition (DDA) to determine the best phosphopeptide(s) with which to represent each phosphorylation site. Specifically, for each phosphorylation site, we determined which charge state and peptide cleavage form was the most frequently observed in the DDA data. Importantly, this database also enabled accurate prediction of the chromatographic retention time. In some cases, phosphorylation sites were represented by multiple peptides in the preliminary target list because it was not obvious which peptide form to choose. To refine the preliminary target list, three separate PRM assays were used to measure these phosphopeptides in insulin-stimulated WT cells with wide (+/−2.5 min) windows for retention time prediction. Peptides that were not detected or contained complete overlap of phosphorylation sites with another detected peptide were removed from the list, and all retention time predictions were re-calibrated. This final target list was then used for PRM analysis of all the samples with narrow (+/−1.5 min) windows for retention time prediction.

Targeted phosphoproteomic analysis of preadipocyte phosphorylation sites was performed using LC-MS/MS. To normalize for injection amount during LC-MS/MS analysis, we measured all samples using DDA and identified 12 phosphopeptides that correlated strongly with the total MS signal and spanned most of the retention time space. DDA analysis was performed on a Thermo QExactive mass spectrometer connected to a Thermo Easy-nLC II. Chromatographic separation was performed on a 30-cm length column with a 100-μm internal diameter, packed with Reprosil 1.9 μm C18 beads (Dr. Maisch GmbH). The LC method consisted of a 49-min linear gradient from 9% to 25% acetonitrile in 0.15% formic acid. Data were acquired throughout a 70-min total time. Full MS scans were acquired from 300 to 1500 *m*/*z* at 70,000 resolution with an AGC target of 3 × 10^6^ and a maximum injection time of 240 ms. The top 20 most abundant precursors per MS scan were isolated with a 2-*m*/*z* window, an AGC target of 2 × 10^5^, a maximum injection time of 120 ms, and 40 s dynamic exclusion. Isolated precursors were fragmented by high-energy collision-induced dissociation at a normalized collision energy of 27%, and MS/MS were acquired at 17,500 resolution. Targeted LC-MS/MS using PRM was performed on the same instrument set-up as for DDA. The instrument methods were mostly the same as for the DDA analysis, except that MS/MS spectra were acquired according to the predefined schedule of precursors representing the phosphopeptide target list, as described above. Every 20 scans, a full MS scan was acquired as described above for DDA. All MS and MS/MS spectra for DDA and PRM experiments were collected in centroid mode. PRM data were analyzed using Skyline^53^, and all chromatographic peaks and phosphorylation site localizations were manually inspected. For additional verification of phosphorylation site localization, PRM data were searched using Comet (v2015.02.5)^54^ and filtered at 1% FDR using Percolator^55^. Each MS/MS spectrum was scored for localization using Ascore^56^. Ascore values representing confidence of site localization were plotted vs retention time and manually compared to the fragment chromatographic elution peaks in Skyline. Phosphorylation sites were stratified into three categories of mTORC2 sensitivity by calculating ANOVA statistics to determine the significant effects of *Rictor* loss and correcting to *q*-values using the Benjamini–Hochberg method. Class I sites had *q* ≤ 0.05 and at least a 2-fold difference between control and *Rictor-iKO*^*PBA*^. Class III sites had *q* ≤ 0.05 and at least a 1.33-fold difference between control and *Rictor-iKO*^*PBA*^. All the phosphoproteomic data generated during this study are available and were deposited in Massive.Ucsd.edu [https://massive.ucsd.edu/ProteoSAFe/dataset.jsp?task=2335fcac3c184be69e7efe068387f771].

### Metabolite analysis

Cells were incubated in serum-free media for 12 h and then incubated for 3 h with fresh DMEM medium. Samples were prepared by performing an 80% methanol extraction and stored at −80 °C until shipping. Polar metabolite profiling methods were developed at the Whitehead Institute for Biomedical Research Metabolomics core facility, where the measurements were performed. Reference standards of each metabolite were used to determine chromatographic retention times and MS multiple reaction monitoring transitions, declustering potentials, and collision energies. All metabolites were normalized to the protein concentration of each sample.

### Chromatin immunoprecipitation analysis

Cells were cross-linked with 1% paraformaldehyde for 15 min at room temperature. The reaction was quenched for 5 min at room temperature by adding 0.125 M glycine. After three washes with 1× PBS, cells were lysed with lysis buffer (1% SDS, 20 mM EDTA pH 8, 50 mM Tris-HCl, pH 8) supplemented with protease and deacetylase (TSA) inhibitors. Lysates were sonicated on ice using a Diagenode Bioruptor^TM^ UCD200 sonicator (for 15 min total at high setting in intervals of 45 s on–20 s off and kept on ice during the whole sonication process). Size of fragments obtained (between 200 and 1200 bp) was confirmed by electrophoresis. Soluble chromatin was collected after centrifugation at 16,000 × *g* at 4 °C for 10 min. Soluble chromatin (1%) was kept as input control. Soluble chromatin was precleared with 2.5 μg/mL of protein-A-Sepharose at 50% at 4 °C in rotation for 1 h and beads were spin down at 5900 × *g* for 3 min at 4 °C. After centrifugation, supernatants were collected and specific antibodies (for total H3: Cell Signaling #9715; and for K27H3Ac: Abcam ab4729) were added. Mixtures were incubated overnight at 4 °C for 8 h in rotation and then incubated for 2 h at 4 °C in rotation with protein-A-Sepharose at 50% (Roche). Beads were collected and washed sequentially at 4 °C for 10 min with wash buffer A (140 mM NaCl, 0.1% SDS, 0.1% deoxycholate, 1% Triton X-100, 1 mM EDTA, and 50 mM Hepes (pH 7.9)), wash buffer B (500 mM NaCl, 0.1% SDS, 1% Triton X-100, 0.1% deoxycholate, 1 mM EDTA, and 50 mM Hepes (pH 7.9.1)), LiCl buffer (20 mM Tris-HCl (pH 8), 1 mM EDTA, 250 mM LiCl, 0.5% deoxycholate, and 0.5% NP-40), and TE buffer (10 mM Tris-HCL (pH*), 1 mM EDTA). Immunoprecipitates were eluted two times with elution buffer elution buffer (50 mM Tris-HCl (pH 8), 1 mM EDTA, and 1% SDS). Reversion of cross-linking was performed overnight by heating samples and input controls at 65 °C. Samples were then treated with 0.2 mg/mL RNAse A (Qiagen) and incubated for 30 min at 37 °C followed by addition of 1 μL of (20 mg/mL) Proteinase K (Promega), and samples were incubated at 45 °C for 1 h. DNA was then purified using the QIAquick Spin Kit (Qiagen). Real-time reverse transcriptase PCR was performed with primers listed in Supplementary Table 1.

### Acetyl-CoA measurements and isotopic tracer analysis

Cells were fasted for 12 h and then incubated in DMEM without glucose plus 5 mM of normal or ^3^C_6_-glucose and 100 μM normal or ^13^C_2_ acetate at 37 °C for 1 h. Samples were harvested in cold PBS, centrifuged 1000 × *g*, 5 min, and the pellet was quenched in 10% (w/v) ice cold trichloroacetic acid in water, and then frozen at −80 °C until extraction and analysis on a QExactive Plus (Thermo) mass spectrometer coupled to an Ultimate 3000 high-performance LC. For quantitation, the abundance of acyl-CoA species was determined by interpolation to a linear standard curve. For isotopic tracer analysis, isotopic enrichment was calculated to compensate for the non-linearity of isotopic enrichment using the FluxFix calculator^57^.

### *De novo* lipogenesis assay

Cells were incubated for 3 days with DMEM in which 0.01% of the total glucose concentration of the medium was comprised of D-[U-^14^C]-glucose. Chloroform extraction was performed, and labeled lipids were measured using a scintillation counter. Each sample was normalized to total protein concentration^58^.

### Lipid staining

Cells were fixed with 4% paraformaldehyde, washed with distilled water, and treated with 100% propylene glycol twice for 5 min followed by a 15-min incubation with Oil Red O solution. Then cells were washed with 85% propylene glycol for 3 min and rinsed twice in distilled water. Oil Red O stain was quantified with the ImageJ software (http://imagej.net/Welcome) and in each case shows the average quantification of four images representing each quadrant of the well.

### Gene expression analysis

Cells or tissues were lysed with Qiazol (Invitrogen) and total RNA was isolated with the RNeasy Kit (Invitrogen). Equal amounts of RNA (2 μg) were retro-transcribed to cDNA using a high-capacity cDNA Reverse Transcription Kit (#4368813, Applied Biosystems). Quantitative real-time PCR was performed in 10 μL reactions using a StepOnePlus real-time PCR machine from Applied Biosystems using SYBR Green PCR master mix (#4309156, Applied Biosystems) according to the manufacturer’s instructions. Relative mRNA expression was determined by the ΔΔCt method and Tbp expression was used as a normalization gene in all conventional PCR with reverse transcription experiments. Primer information is listed in Supplementary Table 1.

### Quantification and statistical analysis

Data are presented as mean + SEM; *p < 0.05, **p < 0.01, ***p < 0.001, ****p < 0.0001, a represents ***p < 0.001 vs control (EV), and b represents ***p < 0.001 vs iKO (EV) unless stated otherwise. Student’s *t* test or two-way ANOVA with the Sidak’s test, as appropriate, was used to determine statistical significance. Statistical analysis was done using GraphPad Prism.

### Reporting summary

Further information on research design is available in the Nature Research Reporting Summary linked to this article.

## Supplementary information

Supplementary Information

Description of Additional Supplementary Information

Supplementary Data 1

Supplementary Data 2

Reporting Summary

## Data Availability

All the phosphoproteomic data generated during this study are available in Massive.Ucsd.edu [https://massive.ucsd.edu/ProteoSAFe/dataset.jsp?task=2335fcac3c184be69e7efe068387f771] and the analysis performed is shown in Supplementary Data 1. All other materials, data, and/or associated protocols will also be made available upon request.
