# Author Correction: mTORC2-AKT signaling to ATP-citrate lyase drives brown adipogenesis and de novo lipogenesis

**DOI:** 10.1038/s41467-020-18510-9

**Published:** 2020-09-08

**Authors:** C. Martinez Calejman, S. Trefely, S. W. Entwisle, A. Luciano, S. M. Jung, W. Hsiao, A. Torres, C. M. Hung, H. Li, N. W. Snyder, J. Villén, K. E. Wellen, D. A. Guertin

**Affiliations:** 1 Program in Molecular Medicine, University of Massachusetts Medical School, Worcester, MA 01605 USA; 2 Department of Cancer Biology, University of Pennsylvania, Philadelphia, PA 19104 USA; 3 Abramson Family Cancer Research Institute, University of Pennsylvania, Philadelphia, PA 19104 USA; 4 AJ Drexel Autism Institute, Drexel University, Philadelphia, PA 19104 USA; 5 Department of Genome Sciences, University of Washington, Seattle, WA 98195 USA; 6 Program in Molecular and Cellular Biology, University of Washington, Seattle, WA 98195 USA; 7 Department of Molecular, Cell and Cancer Biology, University of Massachusetts Medical School, Worcester, MA 01605 USA

**Keywords:** Kinases, Proteomics

Correction to: *Nature Communications* 10.1038/s41467-020-14430-w, published online 29 January 2020.

The original version of this article omitted the following from the “Acknowledgements” section:

A.L. is supported by a post-doctoral fellowship from the American Cancer Society (PF-19-103-01-TBE).

This has now been corrected in both the PDF and HTML versions of the article.

