# Supplementary material for: mTORC2-AKT signaling to ATP-citrate lyase drives brown adipogenesis and de novo lipogenesis
Source: Nat Commun. 2020 Jan 29;11:575. doi: 10.1038/s41467-020-14430-w (PMC6989638; doi:10.1038/s41467-020-14430-w)

Supplementary Information

mTORC2-AKT signaling to ATP Citrate Lyase drives brown adipogenesis and *de novo* lipogenesis

C. Martinez Calejman, S. Trefely, S. W. Entwisle, A. Luciano, S.J. Jung, W. Hsiao, A. Torres, C.M. Hung, H. Li, N. W. Snyder, J. Villén, K. E. Wellen and D. A. Guertin.

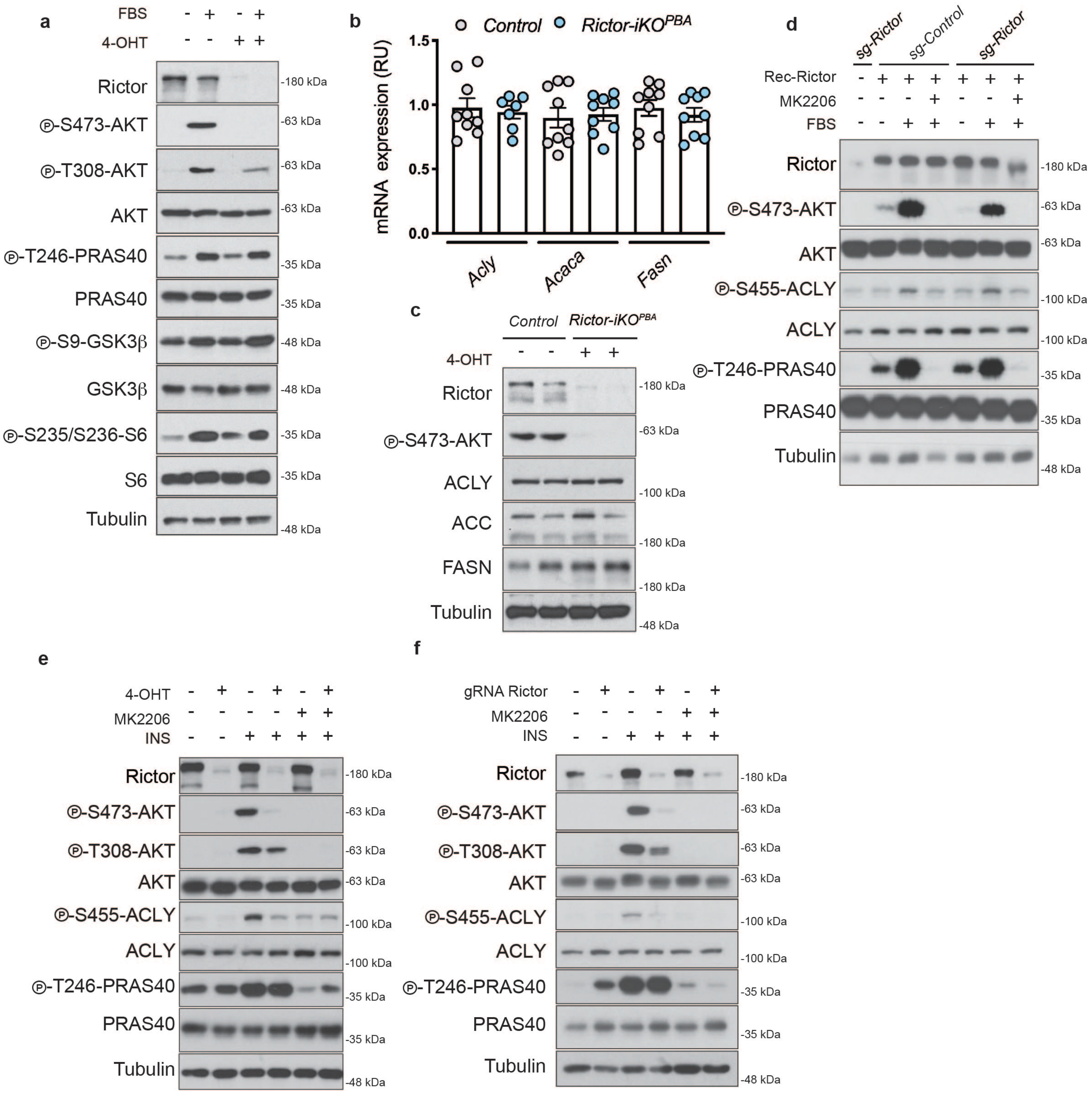

Supplementary Figure 1. Global AKT signaling and DNL gene expression is unaffected by *Rictor* loss in brown preadipocytes (a) Western blots of protein lysates from control and *Rictor-iKO*^{PBA} cells using the indicated total and phospho-specific antibodies. **(b-c)** qRT-PCR analysis (n=7-9) (b) and Western blots (c) of the indicated *de novo* lipogenesis genes from control and *Rictor-iKO*^{PBA} cells**. (d)** Western blots using lysates from HEK293 cells in which recombinant *Rictor* was re-expressed (Rec-Rictor) in cells deleted for *Rictor* by CRISPR/Cas9 genome editing. **(e)** Western blots of protein lysates from control and *Rictor-iKO*^{PBA} cells. Cells growing in DMEM were either serum deprived (-FBS) or serum deprived then stimulated with 150mM of insulin for 15 minutes prior to lysis. Cells were pretreated with or without the AKT inhibitor MK2206 (10μM) for 1 hour. **(f)** Western blots of protein lysates from control and HEK293 cells in which *Rictor* was deleted by CRISPR/Cas9 genome editing and using the indicated total and phospho-specific antibodies. Cells were treated as in (e). Data are mean ± SEM. Statistical significance was calculated using two-way ANOVA with Sidak's test multiple

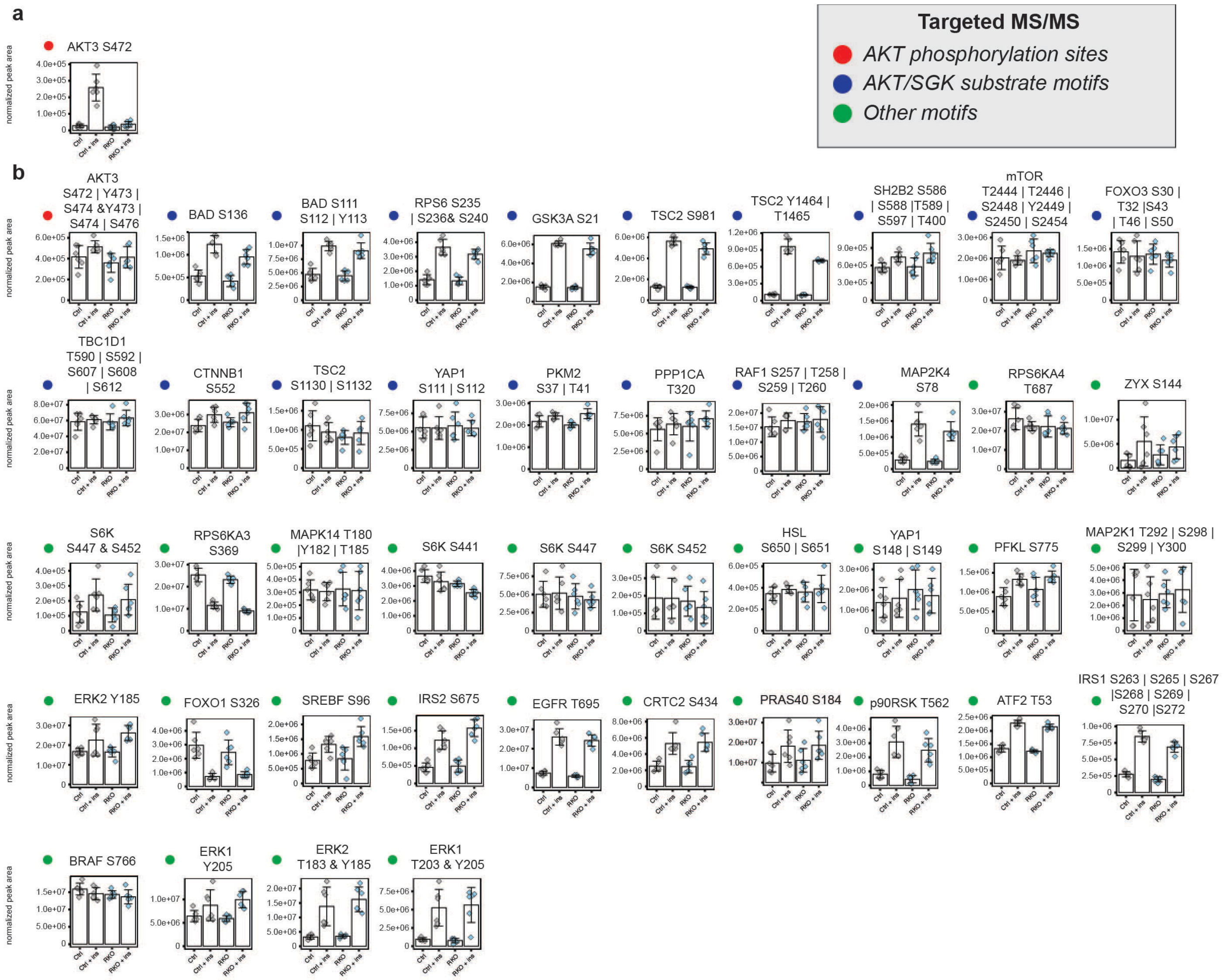

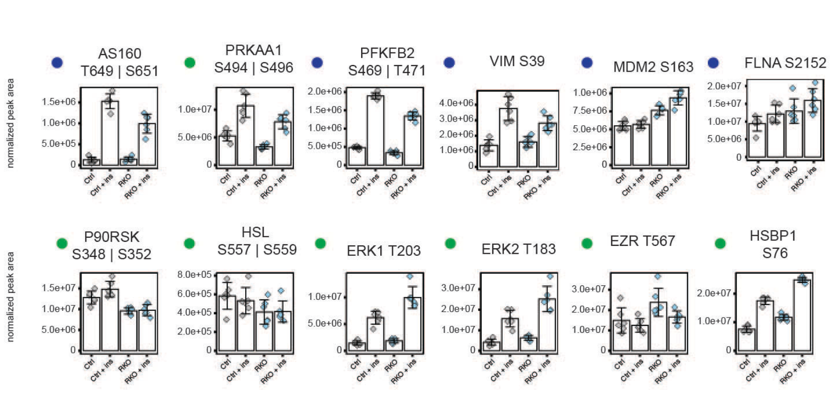

d

Protein	Site	RxRxxS/T motif
**Class III (mTORC2 dependent AKT substrates) ***		
Protein	Site	RxRxxS/T motif
VIMENTIN-S39	TTSTRTYSLGSALR	✖
BAD-S155	RELRRMSDEFEGS	✖
ACLY-S455	APSRTASFSESRA	✖
**Class II (mTORC2 independent AKT substrates)***		
Protein	Site	RxRxxS/T motif
PRAS40-T247	LPRPRLNTSDFQKL	✔
TSC2-S939	SFRARSTSLNERPK	✔
BAD-S136	PFRGRSRSAPPNL	✔
GSK3A-S21	SGRARTSSFAEPG	✔
TSC2-S981	AFRCRSI SVSEHV	✔
MAP2K4-S78	IERLRTHSIESSG	✔

* *Individual phosphosites localized with 95% confidence that increased phosphorylation at least 1.5 fold in response to insulin in the wild -type*

Supplementary Figure 2. Stratification of phosphopeptides based on mTORC2 dependency. Additional phosphopeptides stratified into 3 classes based on mTORC2 sensitivity: **(a)** Class I substrates (highly dependent), **(b)** Class II (insensitive) and **(c)** Class III (partially sensitive). **(d)** Recognition motif analysis on the phospho-sites recognized in our study that show equal or more than 1.5 fold upon insulin stimulation in the control.

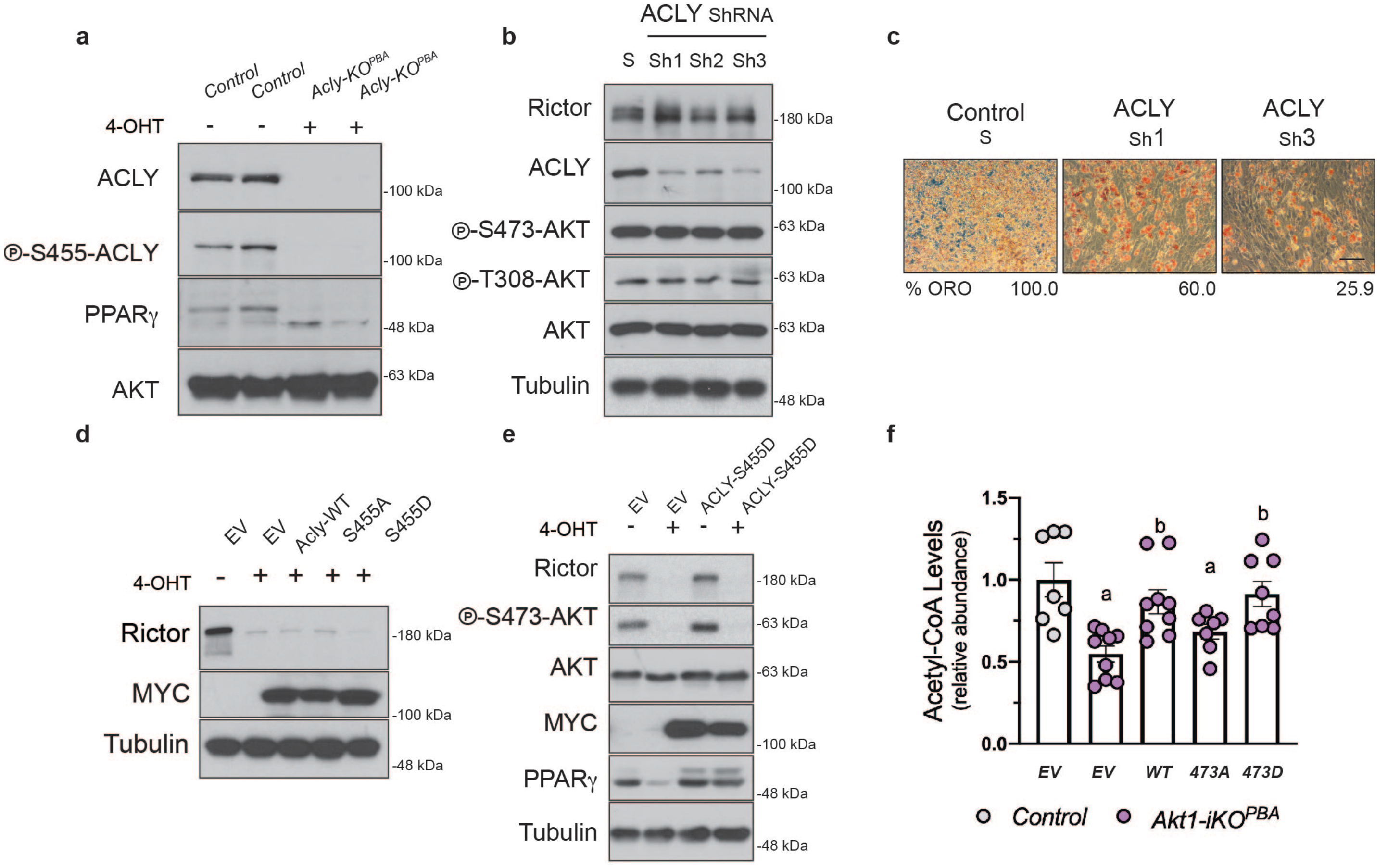

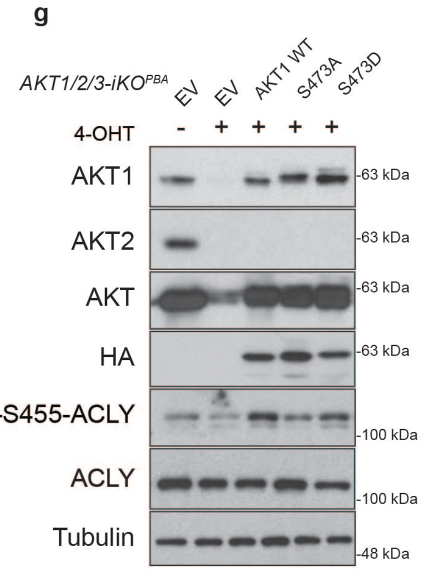

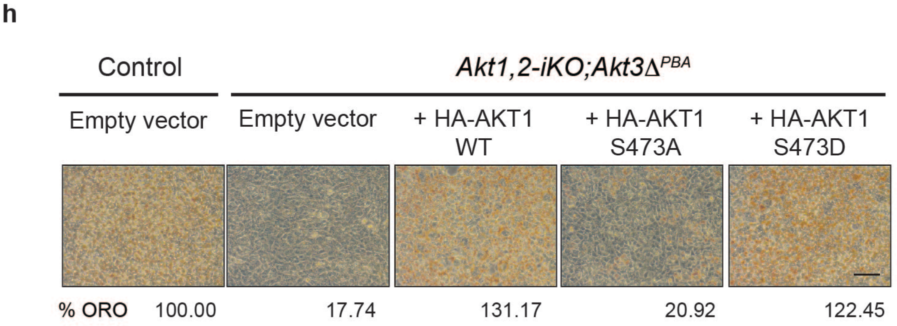

Supplementary Figure 3. Analysis of ACLY knockdown, ACLY-iKO and AKT-triple KO in differentiation assays (a) Western blot of *Acly-iKO*^{PBA} cells on day 10 of differentiation. **(b)** Western blot of Control brown preadipocytes stably transfected with pLKO.1-Scramble (S), pLKO1-ACLY shRNA1(Sh1), pLKO1-ACLY shRNA2 (Sh2) or pLKO1-ACLY shRNA3 (Sh3). Effects of ACLY KD on differentiation were determined by **(c)** Oil Red O stain. Scale bar represents 50 μm. showing stable expression of recombinant of Empty (EV), Myc-ACLY-WT, Myc-ACLY-S455A or Myc-ACLY-455D constructs. **(e)** Western blot of *Rictor-iKO*^{PBA} cells stably expressing Empty (EV) or Myc-ACLY-455D constructs on day 10 of differentiation. (**f**) Direct acetyl-CoA levels (n=7-9) in control and *Akt1-iKO*^{PBA} cells stably expressing Empty (EV), HA-AKT1-WT, HA-AKT1-S473A or HA-AKT1-473D constructs. Bar graphs represent mean ± SEM. a represents $*p < 0.02$ vs control (EV), and b represents $*p < 0.02$ vs *Akt1-iKO*^{PBA} (EV) cells. Statistical significance was calculated by two-way ANOVA with Sidak's test multiple comparisons. **(g-h)** Western blot (d) and Oil red O staining (e) of *Akt1,2-iKO;Akt3Δ*^{PBA} cells stably expressing Empty (EV), HA-AKT1-WT, HA-AKT1-S473A or HA-AKT1-473D constructs.

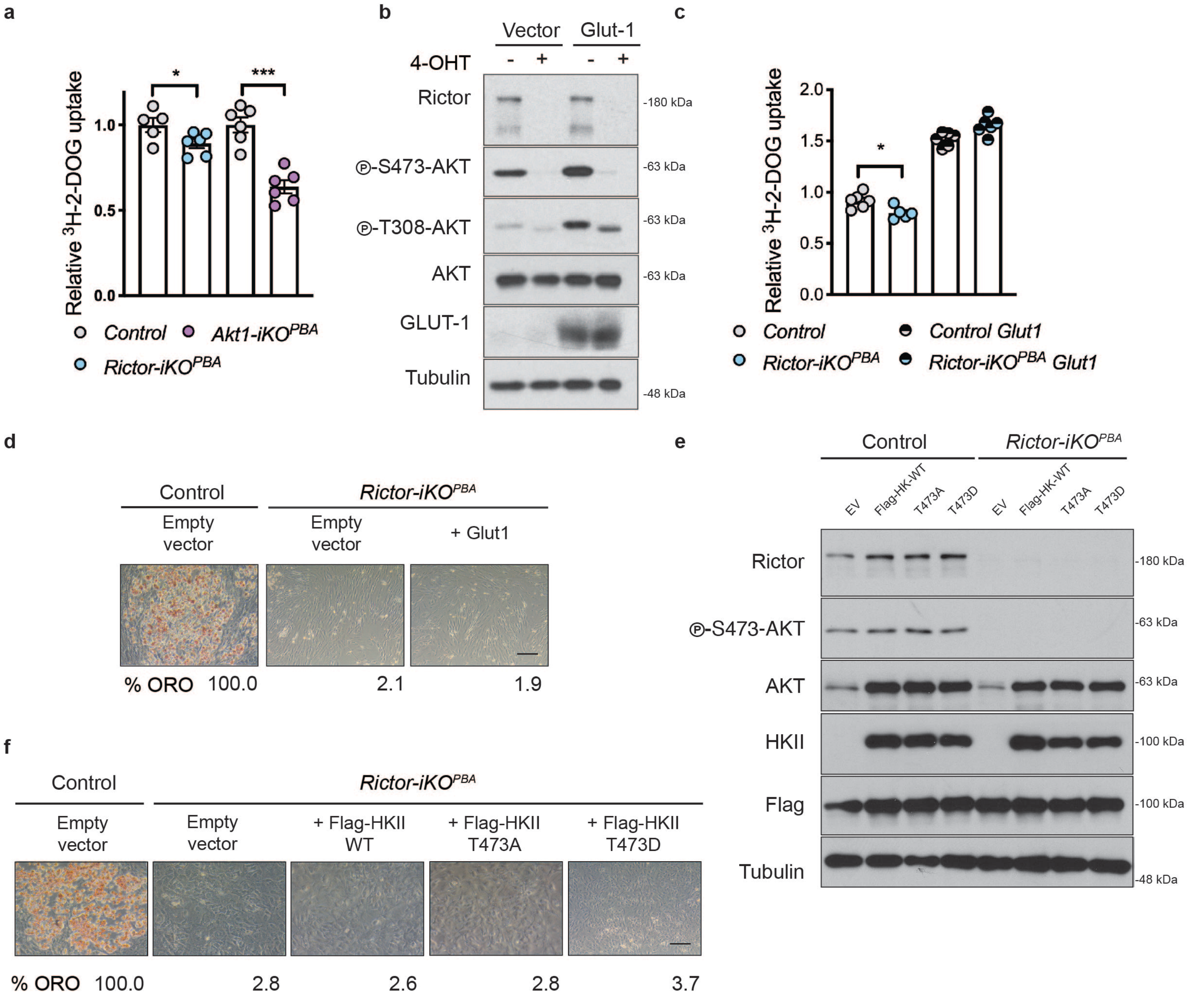

Supplementary Figure 4. Glucose uptake measurements and rescue experiments. (a) Glucose uptake in Control, *Rictor-iKO^{PBA}, or Akt1-iKO^{PBA}* cells (n=6). **(b)** *Rictor-iKO^{PBA}* cells were stably transfected with Empty-pMSCV or pMSCV-Glut-1 vector and Glut-1 expression levels were analyzed by Western blot. **(c)** Glucose uptake measurements (n=6) in of preadipocytes described in (b). Bar graphs represent mean ± SEM. *p < 0.05 and ***p<0.0001. **(d)** Representative differentiation rescue experiments showing and ORO staining. Scale bar represents 50 µm. **(e)** Western blot showing stable expression of Empty-pMSCV or pMSCV-Flag-HK-WT, pMSCV-Flag-HK-T473A or pMSCV-Flag-HK-T473A vector. **(f)** Representative differentiation rescue experiment showing and ORO staining. Scale bar represents 50 µm.Statistical significance was calculated by using two-tailed unpaired Student's t test (a) or two-way ANOVA with Sidak's test multiple comparisons (c).

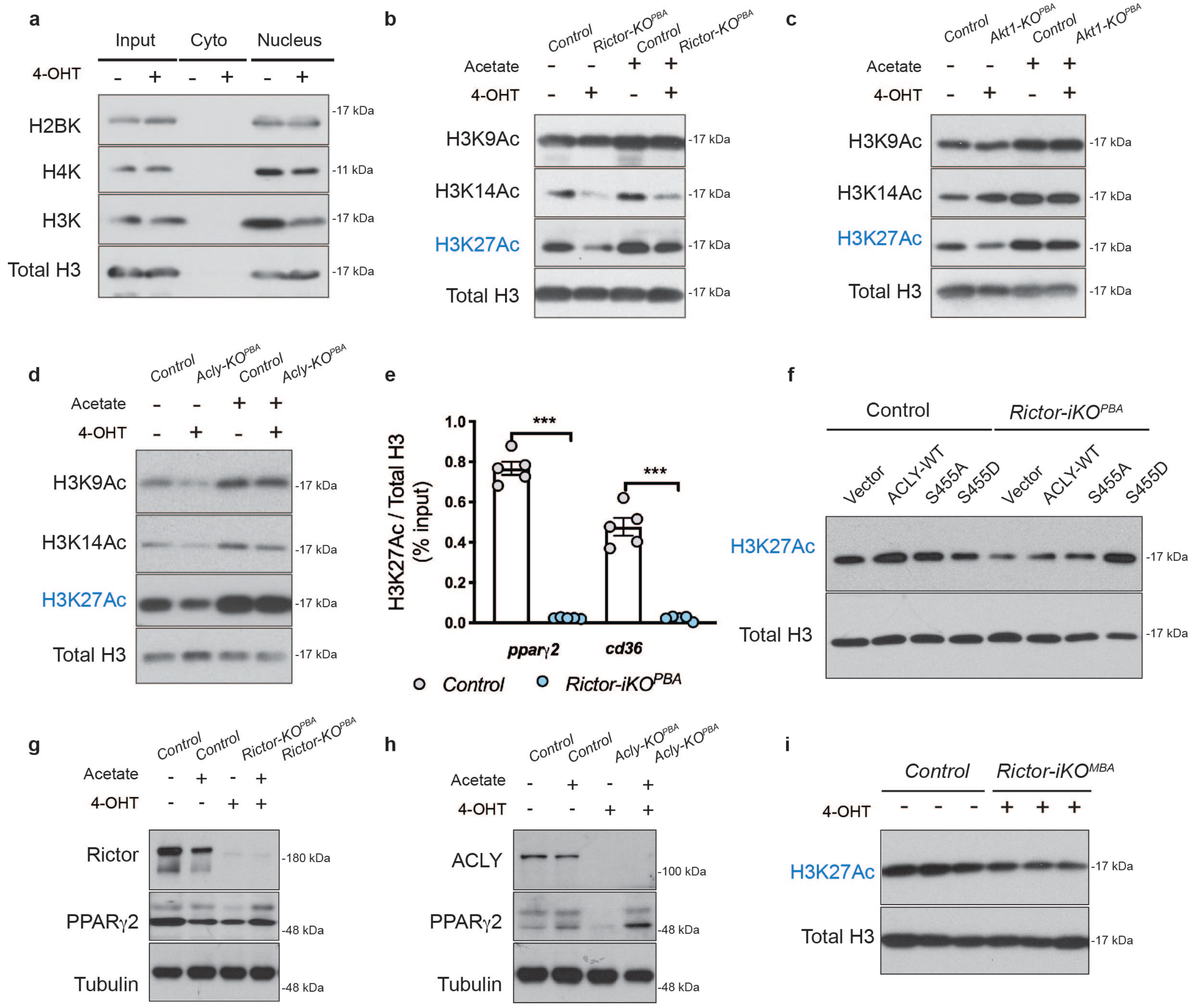

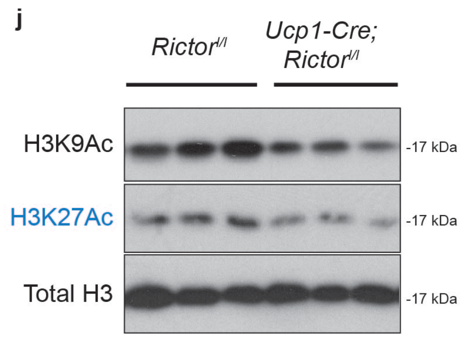

Supplementary Figure 5. Histone acetylation analysis. Western blots of histone analysis marks was performed following chromatin extraction. **(a)** undifferentiated *Rictor-iKO*^{PBA} cells total histone acetylation. **(b-f)** Early differentiating (i.e. day 2) histone analysis. **(a-d)** Selected specific histone marks in *Rictor-iKO*^{PBA} (b), *AKT1-iKO*^{PBA} (c), and *Acly-iKO*^{PBA}cells (d). **(e)** H3K27/Total H3 ChIP analysis of Pparγ2 and CD36 promoter (n=5). Bar graphs represent mean ± SEM. *** p < 0.001 using two-tailed unpaired Student's t test. **(f)** H3K27 acetylation analysis in *Rictor-iKO*^{PBA} cells stably expressing Empty (EV), Myc-ACLY-WT, Myc-ACLY-S455A or Myc-ACLY-455D constructs. **(g-h)** Western blot of *Rictor-iKO*^{PBA} and *Acly-iKO*^{PBA}cells with or without acetate supplementation at day 10 of differentiation. **(I)** H3K27 acetylation in *Rictor-iKO*^{MBA} acutely following induced Rictor deletion (i.e. at day 12 of differentiation). **(j)** H3K27 acetylation in brown fat tissue isolated from control (*Rictor*$^{l/l}$) or *UCP1-Cre-Rictor*$^{l/l}$.

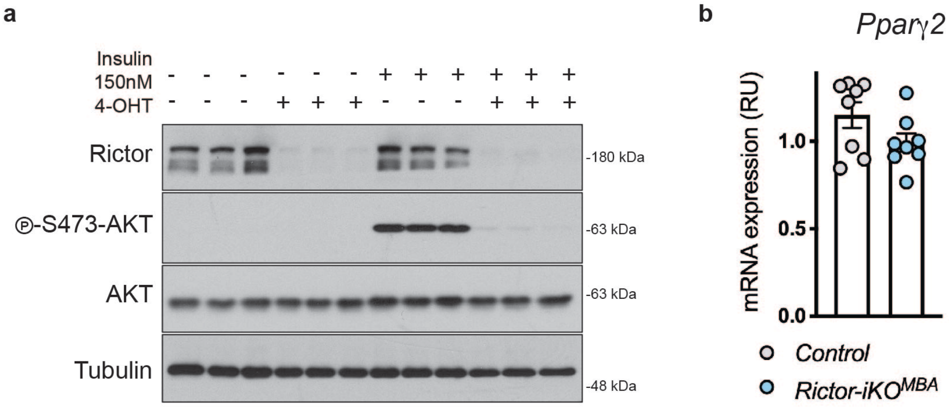

Supplementary Figure 6. Validation of inducible *Rictor* mature brown adipocytes (a) Western blot and **(b)** *Pparγ2* expression of Control and *Rictor-iKO*^{MBA}cells (n=8) following acute *Rictor* loss (i.e. differentiation day 12). Bar graphs represent mean ± SEM. Statistical significance was calculated by using two-tailed unpaired Student's t test

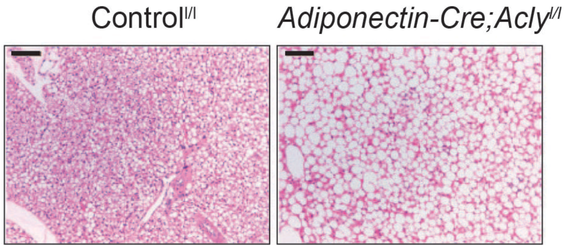

Supplementary Figure 7. BAT morphology in all fat *Acly* KO mice.
Hematoxilin & eosin stain of interscapular BAT of 8 week old control (*Acly*$^{l/l}$) and *Adiponectin-Cre;Acly*$^{l/l}$ mice. Scale bar represents 100 µm.

Gene	Forward primer (5'-3')	Reverse primer (5'-3')
Tbp	GAAGCTGCGGTACAATTCCAG	CCCCTTGTACCCTTCACCAAT
Pparγ2	TCAGCTCTGTGGACCTCTCC	ACCCTTGCATCCTTCACAAG
Chrebpα	CGACACTCACCCACCTCTTC	TTGTTCAGCCGGATCTTGTC
Chrebpβ	TCTGCAGATCGCGTGGAG	CTTGTCCCGGCATAGCAAC
Acly	CTCACACGGAAGCTCCATAA	ACGCCCTCATAGACACCATC
Acaca	GGAGATGTACGCTGACCGAGAA	ACCCGACGCATGGTTTTCA
Fasn	GCTGCGGAAACTTCAGGAAAT	AGAGACGTGTCACTCCTGGACTT
Glut4	GTGACTGGAACACTGGTCCTA	CCAGCCAGTTGCATTGTAG
Pparγ2 *(promoter)*	TGAGGCAGACAGGACTGAAAGTGG	TGGTGCCCATCTGGAAGGCTGC
CD36 *(promoter)*	GCTAAGAAGAGCAGGGACAACA	TCCTTAGCAGAGAGCTTGGCA

Supplementary Table 1: Mouse primer sequences for quantitative RT-PCR analysis.

Scan western blots from main figures

Figure 1

e

Rictor

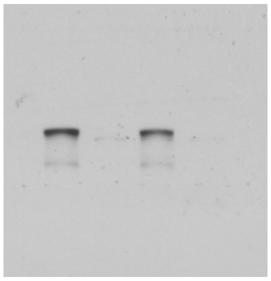

S455-ACLY

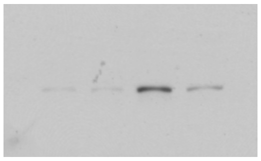

ACLY

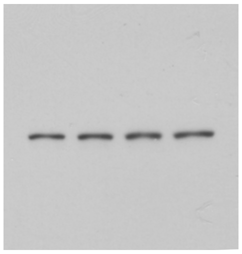

Tubulin

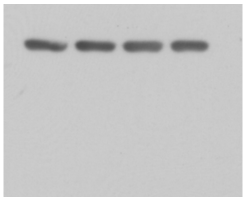

f

Rictor

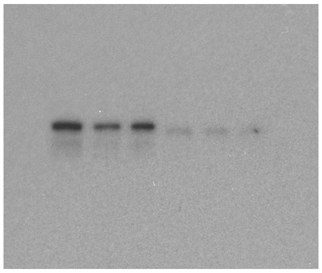

S473-AKT

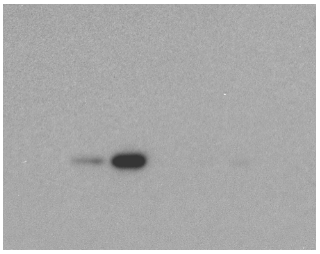

AKT

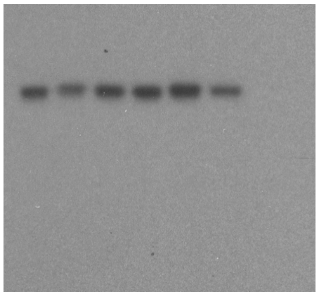

S455-ACLY

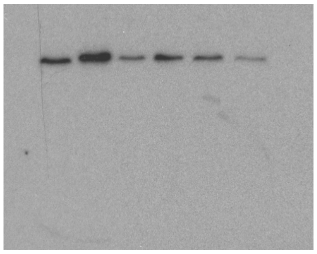

ACLY

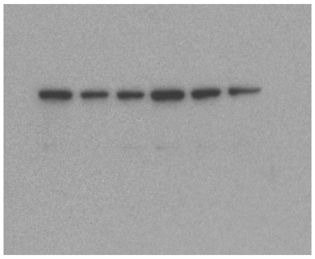

T246-Pras40

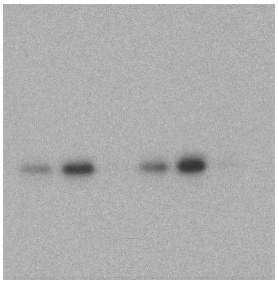

Pras40

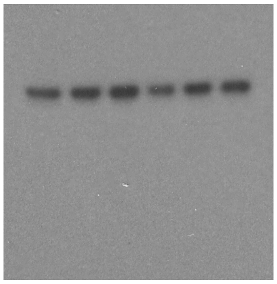

Tubulin

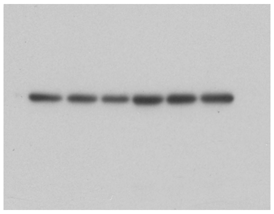

Figure 1
g

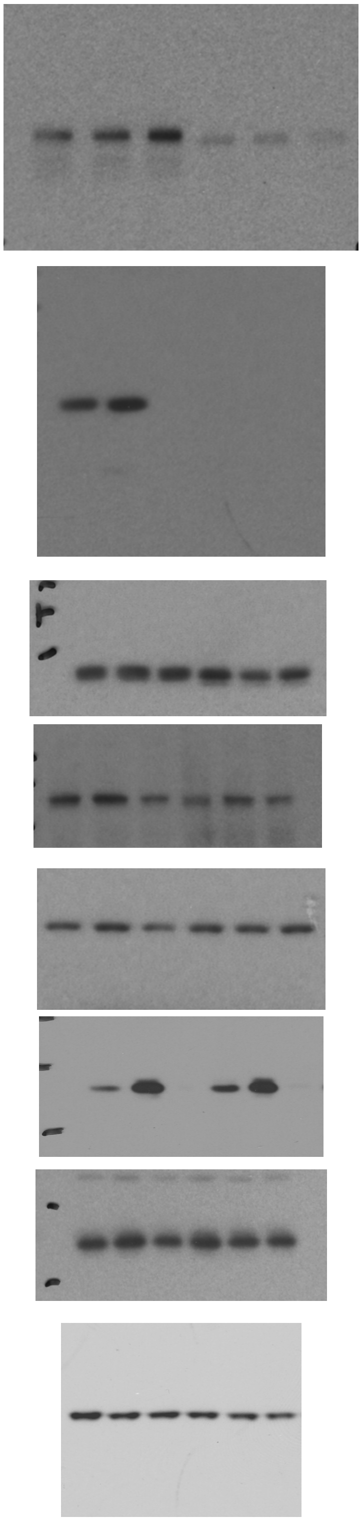

Rictor
S473-AKT
AKT
S455-ACLY
ACLY
T246-Pras40
Pras40
Tubulin

Figure 3
a

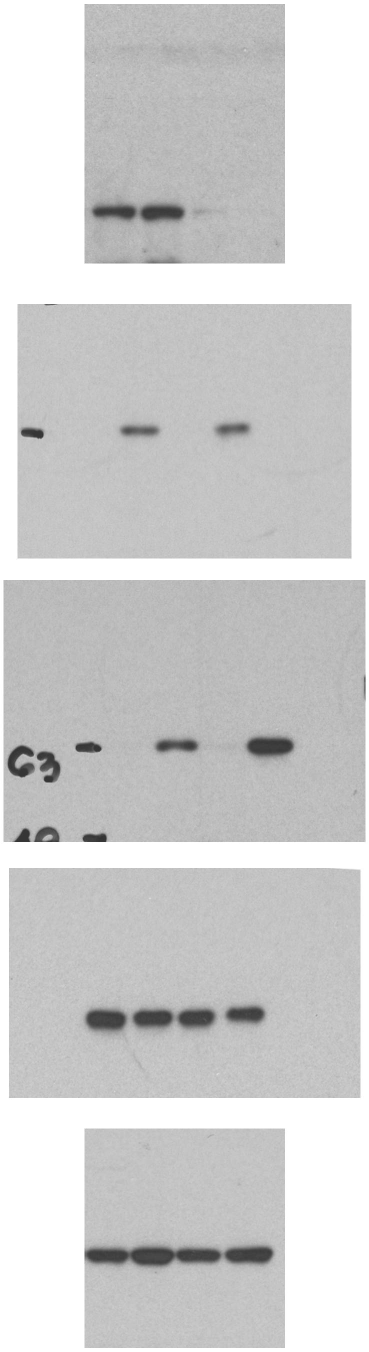

ACLY
T308-AKT
S473-AKT
AKT
Tubulin

Figure 4

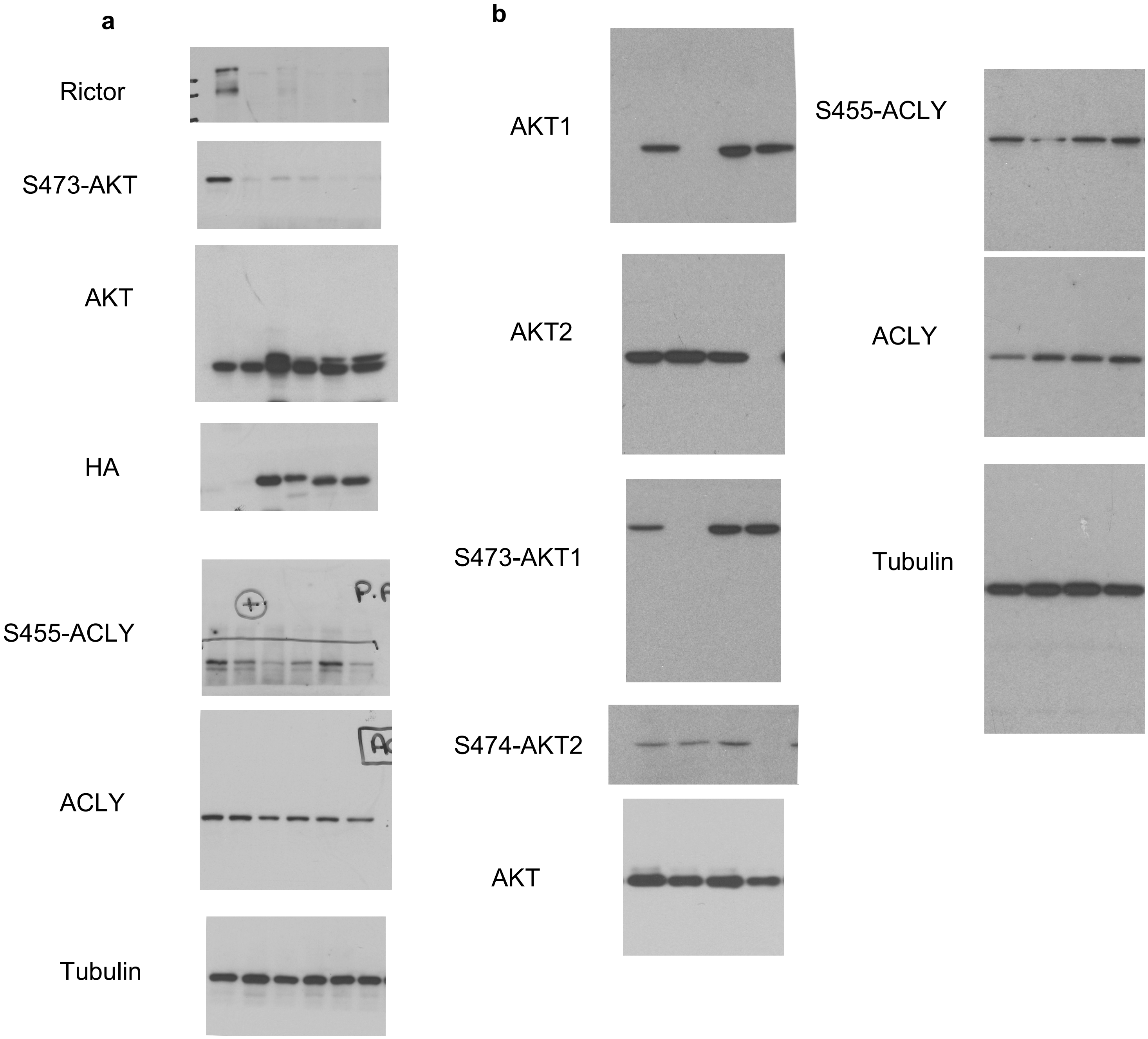
a
Rictor
S473-AKT
AKT
HA
S455-ACLY
ACLY
Tubulin
b
AKT1
AKT2
S473-AKT1
S474-AKT2
AKT
S455-ACLY
ACLY
Tubulin

Figure 4

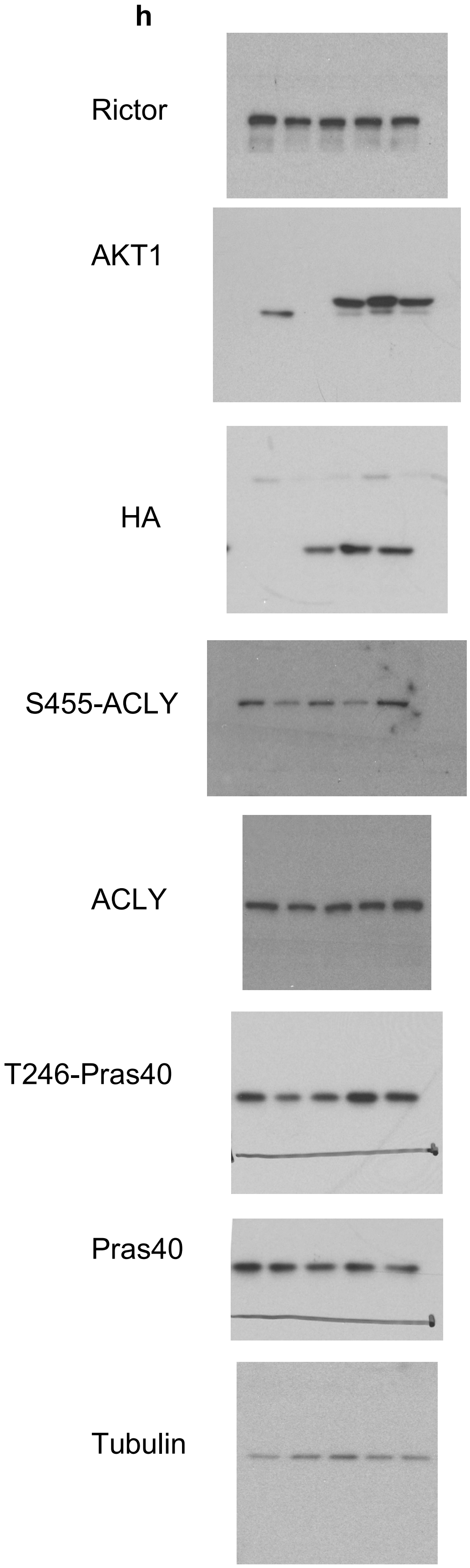

Figure 5

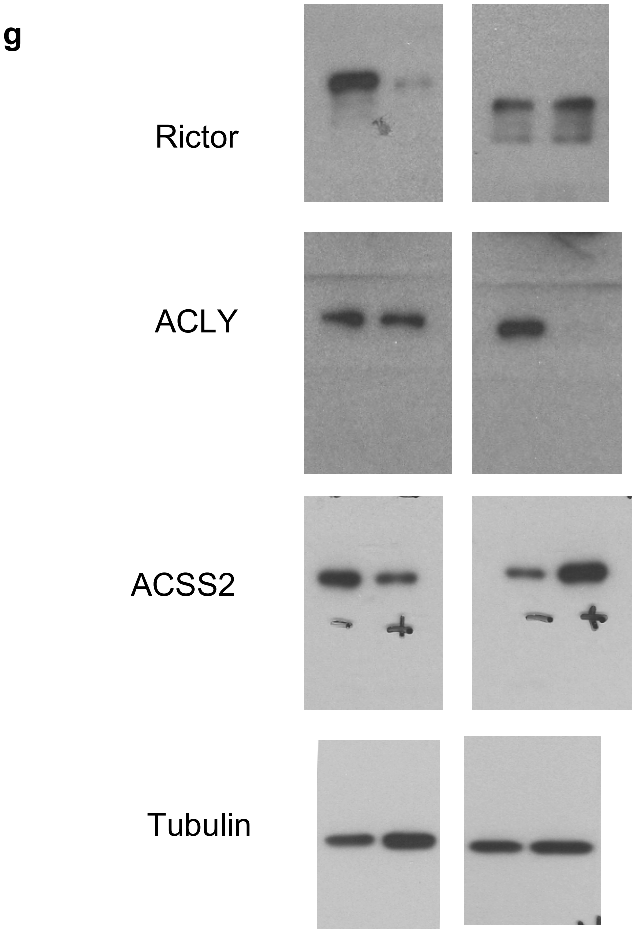

Figure 5

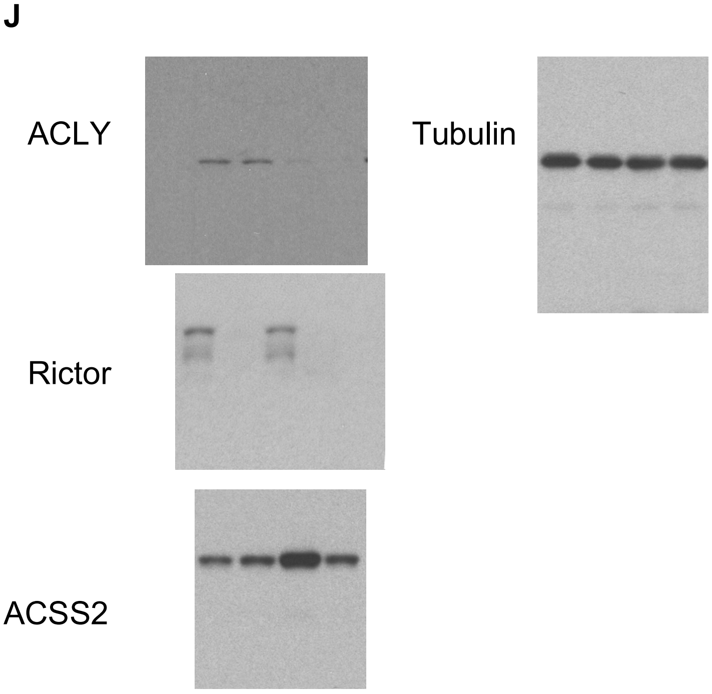

Figure 6

a

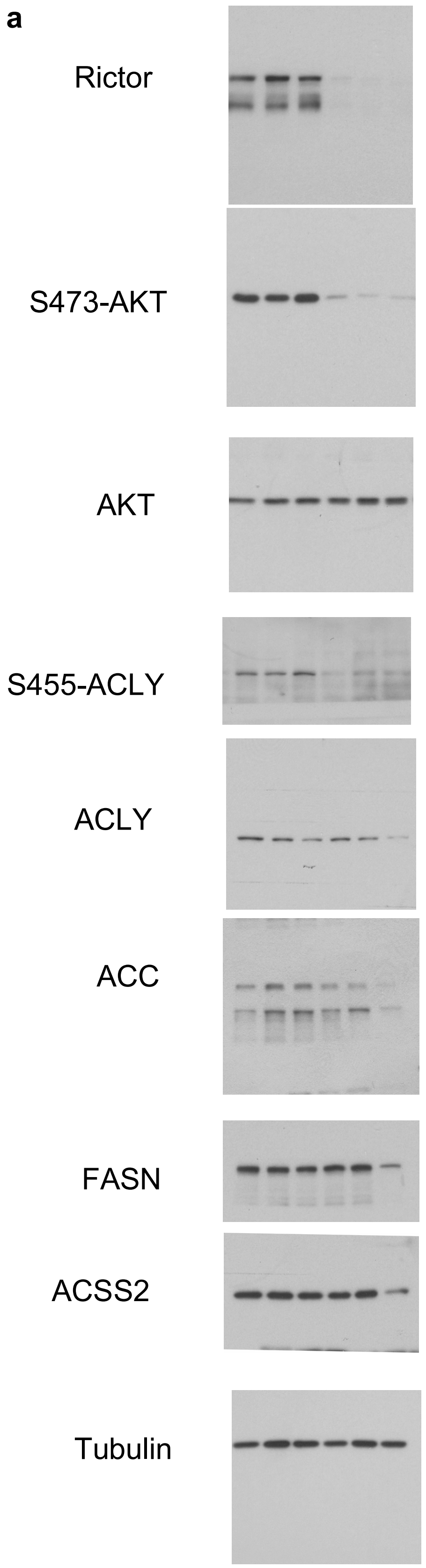

Figure 7

b

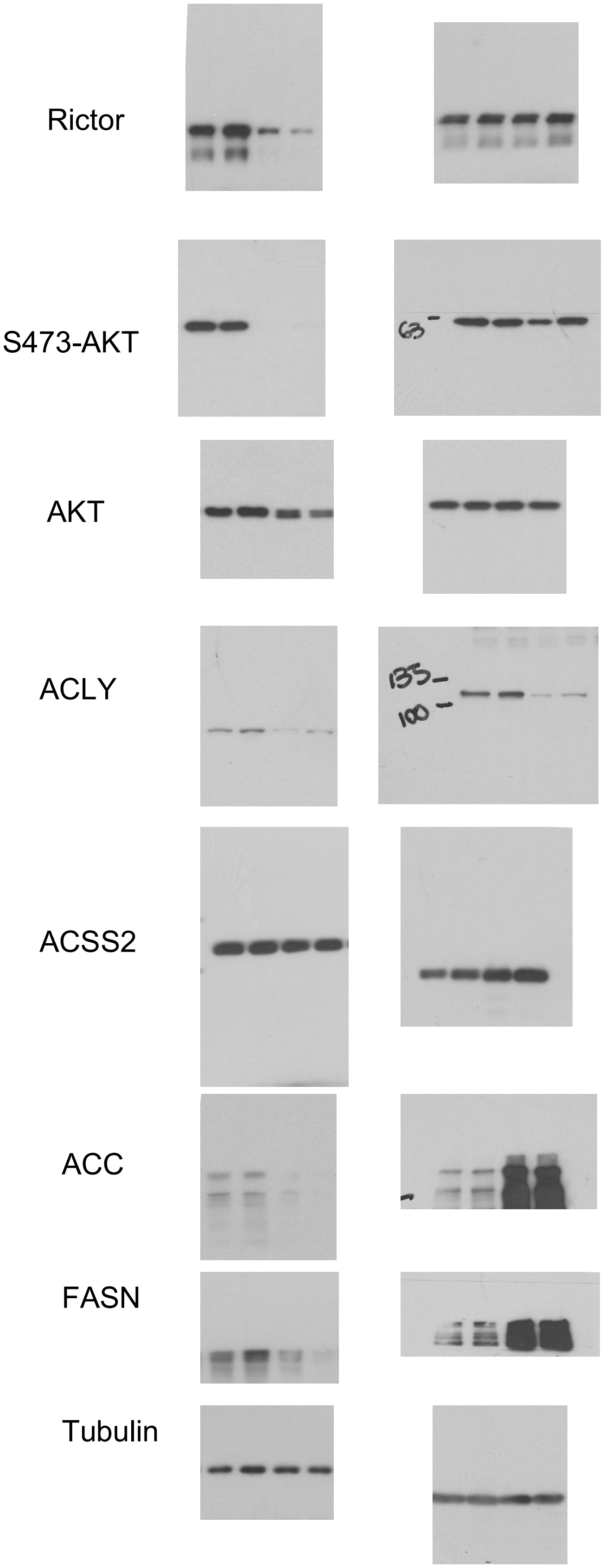

Supplement: Supplementary file 1 — Supplementary Information [file 41467_2020_14430_MOESM1_ESM.pdf]
