# Supplementary material for: mTORC2-AKT signaling to ATP-citrate lyase drives brown adipogenesis and de novo lipogenesis
Source: Nat Commun. 2020 Jan 29;11:575. doi: 10.1038/s41467-020-14430-w (PMC6989638; doi:10.1038/s41467-020-14430-w)
Supplement: Supplementary file 2 — Description of Additional Supplementary Information [file 41467_2020_14430_MOESM2_ESM.docx]

**Description of Additional Supplementary Files**

**File Name**: Supplementary Data 1
**Description:** The supplementary data set 1 contains the data from the phosphoproteomic experiment performed in *Rictor-iKO^PBA^*cells that were serum deprived for 12 hours and treated with or without insulin for 15 minutes.

**File Name**: Supplementary Data 2
**Description:** The supplementary data set 2 contains the data from the metabolomic experiments performed in in *Rictor-iKO^PBA^*cells that were serum deprived for 12 hours and treated with fresh media for 3 h.
