# Supplementary material for: mTORC2-AKT signaling to ATP-citrate lyase drives brown adipogenesis and de novo lipogenesis
Source: Nat Commun. 2020 Jan 29;11:575. doi: 10.1038/s41467-020-14430-w (PMC6989638; doi:10.1038/s41467-020-14430-w)
Supplement: Supplementary file 5 — Reporting Summary [file 41467_2020_14430_MOESM5_ESM.pdf]

nature research

Corresponding author(s): David Guertin

Last updated by author(s): 2019 12 09

Reporting Summary

Nature Research wishes to improve the reproducibility of the work that we publish. This form provides structure for consistency and transparency in reporting. For further information on Nature Research policies, see Authors & Referees and the Editorial Policy Checklist .

Statistics

For all statistical analyses, confirm that the following items are present in the figure legend, table legend, main text, or Methods section.

n/a	Confirmed	
☐	☒	The exact sample size (*n*) for each experimental group/condition, given as a discrete number and unit of measurement
☐	☒	A statement on whether measurements were taken from distinct samples or whether the same sample was measured repeatedly
☐	☒	The statistical test(s) used AND whether they are one- or two-sided<br>*Only common tests should be described solely by name; describe more complex techniques in the Methods section.*
☐	☒	A description of all covariates tested
☐	☒	A description of any assumptions or corrections, such as tests of normality and adjustment for multiple comparisons
☐	☒	A full description of the statistical parameters including central tendency (e.g. means) or other basic estimates (e.g. regression coefficient) AND variation (e.g. standard deviation) or associated estimates of uncertainty (e.g. confidence intervals)
☐	☒	For null hypothesis testing, the test statistic (e.g. *F*, *t*, *r*) with confidence intervals, effect sizes, degrees of freedom and *P* value noted<br>*Give P values as exact values whenever suitable.*
☒	☐	For Bayesian analysis, information on the choice of priors and Markov chain Monte Carlo settings
☒	☐	For hierarchical and complex designs, identification of the appropriate level for tests and full reporting of outcomes
☒	☐	Estimates of effect sizes (e.g. Cohen's *d*, Pearson's *r*), indicating how they were calculated

Our web collection on statistics for biologists contains articles on many of the points above.

Software and code

Policy information about availability of computer code

Data collection	n/a
Data analysis	n/a

For manuscripts utilizing custom algorithms or software that are central to the research but not yet described in published literature, software must be made available to editors/reviewers. We strongly encourage code deposition in a community repository (e.g. GitHub). See the Nature Research guidelines for submitting code & software for further information.

Data

Policy information about availability of data

All manuscripts must include a data availability statement. This statement should provide the following information, where applicable:

- Accession codes, unique identifiers, or web links for publicly available datasets
- A list of figures that have associated raw data
- A description of any restrictions on data availability

all main and supplementary raw data is available

Field-specific reporting

Please select the one below that is the best fit for your research. If you are not sure, read the appropriate sections before making your selection.

☒ Life sciences ☐ Behavioural & social sciences ☐ Ecological, evolutionary & environmental sciences

For a reference copy of the document with all sections, see nature.com/documents/nr-reporting-summary-flat.pdf

Life sciences study design

All studies must disclose on these points even when the disclosure is negative.

Sample size	In all experiments involving animals a sample size between 6 and 8 was used due to the greater biological variability. In cell culture the sample size was between 3 and 6.
Data exclusions	Using GraphPad Prism software, individual technical replicates were excluded from qPCR analysis if they were calculated to be outliers by the "identify outliers" function. No other data were excluded from analyses.
Replication	All experimental findings were replicated at least 3 times
Randomization	Samples and animal subjects were allocated randomly
Blinding	The investigators were blinded at the time of the data acquisition

Reporting for specific materials, systems and methods

We require information from authors about some types of materials, experimental systems and methods used in many studies. Here, indicate whether each material, system or method listed is relevant to your study. If you are not sure if a list item applies to your research, read the appropriate section before selecting a response.

Materials & experimental systems

n/a	Involved in the study	
☐	☒	Antibodies
☐	☒	Eukaryotic cell lines
☐	☐	Palaeontology
☐	☒	Animals and other organisms
☒	☐	Human research participants
☒	☐	Clinical data

Methods

n/a	Involved in the study	
☒	☐	ChIP-seq
☒	☐	Flow cytometry
☒	☐	MRI-based neuroimaging

Antibodies

Antibodies used	GLUT-1 (ab652) and H3K27Ac (ab4729) were purchased from Abcam. HK (A0994) and HRP-goat anti-Rabbit were purchased from ABclonal. HRP-conjugate anti-mouse (w402b) was purchased from Promega. H2BK (07-373), H4K (06-866) and H3K (06-599) were purchased from Millipore. Flag (F7425) was purchased from Sigma-Aldrich. All other antibodies including Rictor (2140), PRAS40 (2691), AKT (9272), GSK3b (9315), ACC (3676), ACLY (4332), FASN (3180), PPARg (2443), ACSS2 (3658), S6 (2217), S473-AKT (4058), T308-AKT (4056), S455-ACLY (4331), T246-pPRAS40 (2997), HA-tag (2367), S9-GSK3b (9315), S235/236-S6 (4858), H3 (9715), H3K9Ac (9649), H3K14Ac (7627), a-Tubulin (2125) were purchased from Cell Signaling Technologies.
Validation	All antibodies were validated in mouse by the correspondent manufacturer.

Eukaryotic cell lines

Policy information about cell lines

Cell line source(s)	iBAT cells were generated from interscapular brown fat tissue and immortalized using SV40 large T virus and 293 T cells were purchased in ATCC
Authentication	All cell lines were authenticated as iBAT cell lines by differentiating them and the presence of iBAT markers by qPCR
Mycoplasma contamination	They were tested for upon generation or acquisition
Commonly misidentified lines (See ICLAC register)	n/a

Palaeontology

Specimen provenance	n/a
Specimen deposition	n/a

Dating methods	n/a

☐ Tick this box to confirm that the raw and calibrated dates are available in the paper or in Supplementary Information.

Animals and other organisms

Policy information about studies involving animals; ARRIVE guidelines recommended for reporting animal research

Laboratory animals	C57BL/6J mice (JAX stock 000664), Ucp1-Cre (JAX stock 024670), Adiponectin-Cre (JAX stock 010803), Rictorl/l and Aclyl/l mice. All animals involved in this study were 8 week-old males
Wild animals	n/a
Field-collected samples	n/a
Ethics oversight	Umass Medical School Committee on Animal Care (IACUC).

Note that full information on the approval of the study protocol must also be provided in the manuscript.
